# Biomimetic natural biomaterials for tissue engineering and regenerative medicine: new biosynthesis methods, recent advances, and emerging applications

**DOI:** 10.1186/s40779-023-00448-w

**Published:** 2023-03-28

**Authors:** Shuai Liu, Jiang-Ming Yu, Yan-Chang Gan, Xiao-Zhong Qiu, Zhe-Chen Gao, Huan Wang, Shi-Xuan Chen, Yuan Xiong, Guo-Hui Liu, Si-En Lin, Alec McCarthy, Johnson V. John, Dai-Xu Wei, Hong-Hao Hou

**Affiliations:** 1 Guangdong Provincial Key Laboratory of Construction and Detection in Tissue Engineering, The Fifth Affiliated Hospital, School of Basic Medical Science, Southern Medical University, Guangzhou, 510900 China; 2 Department of Orthopedics, Tongren Hospital, Shanghai Jiao Tong University, Shanghai, 200336 China; 3 The Eighth Affiliated Hospital, Sun Yat-Sen University, Shenzhen, 518033 Guangdong China; 4 Engineering Research Center of Clinical Functional Materials and Diagnosis & Treatment Devices of Zhejiang Province, Wenzhou Institute, University of Chinese Academy of Sciences, Wenzhou, 325011 Zhejiang China; 5 Department of Orthopedics, Union Hospital, Tongji Medical College, Huazhong University of Science and Technology, Wuhan, 430022 China; 6 Department of Orthopaedics and Traumatology, Faculty of Medicine, the Chinese University of Hong Kong, Hong Kong SAR, 999077 China; 7 Department of Functional Materials, Terasaki Institute for Biomedical Innovation, Los Angeles, CA 90064 USA; 8 Mary & Dick Holland Regenerative Medicine Program, College of Medicine, University of Nebraska Medical Center, Omaha, NE 68130 USA; 9 Zigong Affiliated Hospital of Southwest Medical University, Zigong Psychiatric Research Center, Zigong Institute of Brain Science, Zigong, 643002 Sichuan China; 10 Key Laboratory of Resource Biology and Biotechnology in Western China, Ministry of Education, School of Medicine, Department of Life Sciences and Medicine, Northwest University, Xi’an, 710127 China

**Keywords:** Biomimic, Scaffold, Biosynthesis, Natural biomaterial, Tissue engineering

## Abstract

Biomimetic materials have emerged as attractive and competitive alternatives for tissue engineering (TE) and regenerative medicine. In contrast to conventional biomaterials or synthetic materials, biomimetic scaffolds based on natural biomaterial can offer cells a broad spectrum of biochemical and biophysical cues that mimic the in vivo extracellular matrix (ECM). Additionally, such materials have mechanical adaptability, microstructure interconnectivity, and inherent bioactivity, making them ideal for the design of living implants for specific applications in TE and regenerative medicine. This paper provides an overview for recent progress of biomimetic natural biomaterials (BNBMs), including advances in their preparation, functionality, potential applications and future challenges. We highlight recent advances in the fabrication of BNBMs and outline general strategies for functionalizing and tailoring the BNBMs with various biological and physicochemical characteristics of native ECM. Moreover, we offer an overview of recent key advances in the functionalization and applications of versatile BNBMs for TE applications. Finally, we conclude by offering our perspective on open challenges and future developments in this rapidly-evolving field.

## Background

Tissue engineering (TE) aims to restore, preserve, or enhance the structure and function of defective tissues or organs by integrating biological cues and bioscaffold strategies [1–3]. Bioscaffolds provide a niche for cells by imitating the composition, structure and properties of the in vivo extracellular matrix (ECM) and offer cells a broad spectrum of biological and physicochemical cues. The ECM acts as a biomass network that combines softness, toughness and elasticity to provide mechanical support and structural integrity to tissues and organs. It is mainly composed of a polysaccharide matrix with a variety of embedded proteins, such as collagen, elastin, and fibronectin. The ECM’s three-dimensional (3D) hierarchical microstructure and electromechanical nature play an essential role in its transport properties, cellular communication, mechanotransduction, and growth factor signaling by interacting with cell surface receptors, as well as binding growth factors and other signaling molecules.

Natural biomaterials derived from renewable resources, such as plants, animals and microorganisms, exhibit a large diversity of unique yet complex constituents, microstructures, and physiological properties. Such materials offer a biological support suitable for cell attachment and growth with a diverse set of functions in their native setting [2, 4]. Thus, natural biomaterials, when repopulated with autologous or genetically engineered cells, can serve as the ideal template for the design of living implants for specific applications in TE and regenerative medicine. Consequently, they are a good choice for biomimetic TE scaffolds due to their shape and mechanical adaptability, microstructure interconnectivity and inherent bioactivity, which mimics the native ECM. In addition, these biomimetic natural biomaterials (BNBMs) possess well-defined molecular structures and plentiful active sites enabling further functional modification and/or anchoring with other materials, enabling the preparation of an overwhelming variety of customized products with desirable properties and fine-tuned functions.

However, natural materials face several limitations, such as batch variability, rapid degradation, weak mechanical properties and limited processability, which slow their clinical translation. To mimic the natural ECM, BNBMs-based scaffolds can be tailored to provide superior physicochemical, mechanical and biological properties, thus supporting cell infiltration, adhesion, differentiation, as well as the transport of oxygen and nutrients.

This review provides an overview of the recent progress in the application of BNBMs for TE, including advances in their preparation, functionality, potential applications and future challenges. Several excellent reviews have been published on biomimetic materials [1–3] or natural biomaterials [4–6], but this is a rapidly-emerging field, and new summaries are always in demand. In addition, past reviews have not focused on the biomimetic strategies from the perspective of composition, structure, and performance. By contrast, the present review provides an inclusive outline of the up-to-date BNBMs for TE. In the remainder of this review, we provide a brief introduction of various BNBMs and highlight recent advances in their fabrication. Then we discuss how controllable alteration of BNBMs with a variety of substantial ECM-mimicking properties, such as electrical activity, biomechanical adaptiveness, or stimulus responsiveness, can be developed and employed to endow natural biomaterials with additional possibilities, leading to further therapeutic effects. Additionally, we review the methodologies used to create various functional natural biomaterials, with a specific emphasis on biomimetic approaches and microstructural hydrogels for cell orientation. Finally, we outline the functionalization of BNBMs for TE applications. We conclude the review with an outlook on the application of BNBMs in TE.

## Biosynthesis and engineering of natural biomaterials

**Fig. 1:**
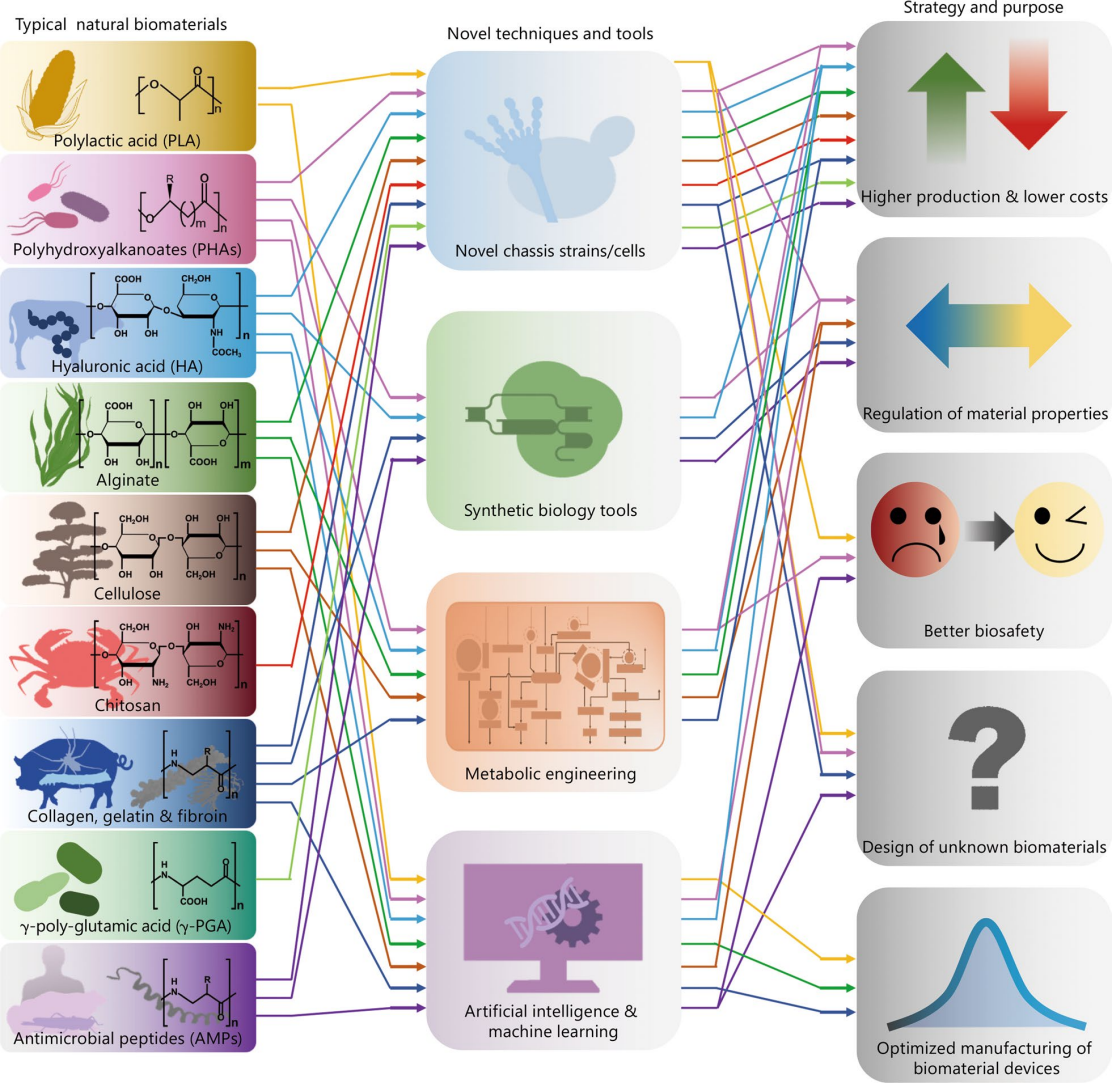
Correlation diagram of multiple strategies and purposes of natural biomaterials for tissue engineering (TE) and regenerative medicine based on novel techniques and tools in recent years. Typical natural biomaterials include biopolyesters of polylactic acid (PLA), polyhydroxyalkanoates (PHAs) and their derivatives (e.g., PLGA, PHA-PLA); polysaccharides of hyaluronic acid (HA), alginate, cellulose and chitosan, proteins and poly (amino acid)s of collagen, gelatin, fibroin, γ-poly-glutamic acid (γ-PGA) and antimicrobial peptides (AMPs), respectively. Novel techniques and tools contain novel chassis strains/cells for biosynthesis, synthetic biology tools, metabolic engineering, artificial intelligence and machine learning, respectively. The strategies for optimized natural biomaterials are higher production and lower costs, regulation of material properties, better biosafety, design of unknown biomaterials, and optimized manufacturing of biomaterial devices, respectively. PLGA poly (lactic-co-glycolic acid), PHA-PLA polyhydroxyalkanoate-polylactic acid

BNBMs are used extensively in TE due to their nontoxicity, non-genotoxicity and non-teratogenicity to native healthy tissue, and excellent bioactivity including promotion of cell proliferation, adhesion, migration and mediation of cell differentiation. The main BNBMs used in TE and regenerative medicine include 1) biopolyesters such as polylactic acid (PLA), polyhydroxyalkanoates (PHAs) and their derivatives, 2) polysaccharides such as hyaluronic acid (HA), alginate, cellulose and chitosan, as well as 3) polypeptides and proteins such as collagen, gelatin, fibroin, poly-glutamic acids (PGA) and antimicrobial peptides (AMPs). The molecular structures of these representative BNBMs are shown in Fig. 1.

### Biopolyesters based BNBMs

PLA is a highly versatile, biodegradable and biocompatible aliphatic polyester. PLA is one of the most widely used biopolyesters and is often applied for the repair of various tissues or deployed for drug delivery. Pure PLA is generally obtained via chemical ring-opening polymerization of lactide, which in turn is commonly derived from lactic acid (LA) produce through microbial fermentation or extraction of materials with high sugar content (such as molasses, whey, bagasse or potato and other cellulose and starch-containing materials) [7, 8]. To regulate the rapid degradation of LA and avoid inflammation due to acidification, glycolide was introduced into PLA to form poly (lactic-co-glycolic acid) (PLGA). Studies have shown that a higher ratio of glycolide units resulted in a more hydrophilic polymer with increased degradation rates [9–11]. In addition, PLA (or its derivatives with a higher molecular weight) showed greater mechanical strength and slower degradation, making them excellent candidates for hard tissue repair, such as 3D printed TE scaffolds for bone and spinal injury [12, 13]. Interestingly, they have also been prepared as fibers for the repair of skin and other soft tissues, as well as microspheres for minimally invasive treatment [14, 15].

Different from PLA and its derivatives, PHAs, another bio-polyester with similar chemical structure (Fig. 1), can only be biosynthesized by microorganisms, including natural and engineered bacteria [16, 17]. Due to their excellent biodegradability, biocompatibility, non-teratogenicity and non-carcinogenicity, various PHAs have been incorporated into a series of biomedical devices for tissue repair and drug delivery. For example, some PHAs have been developed into injectable stem cell carriers [18], 3D in vitro cell culture carriers [19] bone TE scaffolds, nano-carriers for anti-osteoporosis therapy in microgravity [20], as well as for directional bone induction [21], immunoregulation [22] and bionic COVID-19 vaccines [23]. The multiple structures and compositions of PHAs result in a range of mechanical properties and biodegradation rates, as well as PLA with relatively uniform characteristics, which can be adapted to the mechanical and biochemical microenvironments of different tissues. In contrast to faster LA (pKa = 3.86) release from PLA or PLGA, PHAs generally have slow biodegradation and release moderately acidic biodegradable monomers and oligomers of 3-hydroxybutyrate (3HB, pKa = 4.41). Notably, 3HB is a natural metabolite that is present in human blood at concentrations of 0.03–0.1 mg/ml and exhibits a mild irritant effect that diminishes acid load, thereby reducing cellular and tissue reactions resulting in limited local inflammation.

### Polysaccharides based BNBMs

HA is a non-sulfated glycosaminoglycan and a highly hydrated polyanionic macromolecule, which is an essential component of the ECM and mediates its function in tissue repair, signaling, and morphogenesis [24]. Due to the hydrophilic properties of this highly negatively charged polysaccharide, HA can imbibe water to achieve considerable mechanical robustness. As an essential component of the endogenous or synthetic ECM, HA has been proven to be effective at inducing cellular proliferation [25, 26]. In contrast to animal tissue, which has potential pathogenicity and limited sources, HA is easily synthesized on a large scale using various engineered bacteria such as *Lactococcus lactis* [27], *Pichia pastoris* [28] and *Streptococcus* [29], which avoided the residue of pathogens in animals. As a consequence, HA-related products have been widely applied in the pharmaceutical, cosmetic and food industries [30–33].

Alginate, composed of β-D-mannuronic and α-L-guluronic acid residues, is mainly found in brown seaweed and has been included in the U.S. Pharmacopoeia since 1938 [34, 35]. In addition to the food industry, alginates are currently used in TE due to their excellent hydrophilicity, biosafety and inexpensiveness [36–38]. Alginate scaffolds can provide a long-term cell culture support [39] and flat alginate fibers with grooves support myoblast attachment and spreading [40, 41]. Biosynthesis of bacterial alginates using *Pseudomonas aeruginosa* (*P. aeruginosa*) or other microbes is possible, but commercially available alginates are currently derived only from algae [42].

Cellulose is a linear polysaccharide consisting of a chain of several hundred to many thousands of (1 → 4)-β-glucopyranose units, with cellobiose as the dimeric repeating unit [43]. As an important structural component of green plants [44, 45] and some microorganisms [46], it is the most abundant biomaterial on earth. Cellulose is odorless, hydrophilic, biodegradable, biocompatible, and insoluble in water and most organic solvents. The amorphous parts of cellulose are biodegradable in the human body and can be used to regulate the degradation rate of TE scaffolds. In addition, bacterial cellulose (BC) has high water retention and elasticity. It has been approved by the Food and Drug Administration for the clinical usage in wound dressings with incomplete degradation [47]. In contrast to cellulose derived from plant and bacteria, cellulose of animal origin (such as sea squirt cellulose) is more attractive due to its molecular weight, mechanical properties, water holding capacity, water and air permeability and thermal stability [48].

Chitosan is an analogous structure of glycosaminoglycan prepared by partial deacetylation of chitin [49]. Chitosan contains positive charges (zeta potentials of + 20– + 40 mV) and shows potential antibacterial ability, cell adhesion activity and low toxicity. Pure chitosan has been fabricated into nanoparticles, films, sponges, fibers, scaffolds and hydrogels, all of which are commonly used for tissue regeneration and construction [50, 51]. Chitosan-based nano-carriers have been developed for drug delivery in cancer therapy [52, 53]. The introduction of chitosan into a biopolymeric scaffold (i.e., PLA/chitosan scaffolds) results in improved mechanical and biological functionality [54]. On the other hand, chitosan provides better stability, mechanical strength, and biocompatibility in diverse composite scaffolds of proteins or keratin [41, 55, 56].

### Polypeptides and proteins based BNBMs

Collagen consists of a triple helical region composed of three polypeptide strands and two non-helical regions at both ends of the helix [57, 58]. It can be extracted on a large scale from diverse animal by-products such as cattle hides and pigskin. In addition to extraction from animal tissues, a recombinant production system based on yeast has been recently explored [59–62]. Some monodisperse gelatin-like polymers have also been obtained from recombinant protein biosynthesis systems based on engineered microorganisms. Gelatin is another combination of heterogeneous, animal-derived peptides that have a net charge based on environmental pH and gelatin type [59]. Fibroin, which mainly contains repeating amino acid sequences of Gly-Ala-Gly-Ala-Gly-Ser and Gly-Ala-Gly-Ala-Gly-Tyr [60], is also an extensively studied insoluble protein present in silk from silkworms and spiders [61]. A variety of collagen, gelatin and fibroin devices have been developed and applied in medical, pharmaceutical and cosmetics applications for more than a century [62].γ-poly-glutamic acid (γ-PGA) is an anionic polypeptide that has been applied in medicine due to its good water-solubility, biocompatibility, biodegradability and edible properties [63, 64]. Production of γ-PGA relies almost entirely on *Bacillus* species, but the high cost of microbial fermentation processes limits its widespread application in the industry. Recently, several approaches have been exploited to improve the fermentation processes, including novel wildtype strains, recombinant strains, as well as optimization of the culture media and fermentation conditions [65]. For example, we reported a metabolically engineered *Corynebacterium glutamicum* (*C. glutamicum*), which was capable of producing up to 11.4 g/L of γ-PGA from glucose [66]. Thus, the production cost of γ-PGA is greatly reduced, which provides the possibility of its wide application.

AMPs are a class of active oligopeptides with positive charges and amphiphilic structures [67] that have broad-spectrum activity against bacteria, fungi, viruses and parasites, and influence the host immune responses [68, 69]. Natural AMPs are found in microorganisms, plants, invertebrates, fish, amphibians, reptiles, birds and mammals [70]. However, most AMPs are currently biosynthesized for AMP-BioDesign 1.0 by relying on engineered microbial chassis cells [71]. AMPs can increase the permeability of the cell membrane and then destroy its homeostasis, resulting in the cell lysis of targeted pathogens. Some AMPs are transported without damaging the cell membrane and interfere with important targets inside the cell [71].

### BNBMs by novel biosynthesis methods

The properties of BNBMs are difficult to alter because the natural biosynthetic mechanisms are difficult to elucidate, imitate, or adjust. With the development of new technologies, such as novel chassis strains/cells for biosynthesis, synthetic biology, metabolic engineering, artificial intelligence (AI) and machine learning (ML), the large-scale production of BNBMs with a lower cost, tunable properties and better biosafety can be realized by reprogramming in natural or engineered biosynthesis and should also enable the design of novel previously unknown materials by combining desired domains in vivo and with natural templates.

#### Novel chassis strains/cells for BNBMs

Common model microbial chassis cells include *Saccharomyces cerevisiae* (*S. cerevisiae*)*, Escherichia coli* (*E. coli*)*, Bacillus subtilis* (*B. subtilis*) and *Corynebacterium glutamicum* (*C. glutamate*), which have been used to biosynthesize LA (i.e., PLA precursor), PHAs, HA, alginate, cellulose, chitosan collagen, fibroin, γ-PGA and AMPs. The synthesis of these biomaterials by microorganisms avoids the problems of traditional extraction from animal tissues, such as pathogens clearance. Using genetic tools, some non-model microorganisms have been employed as novel chassis strains/cells to produce BNBMs. For example, *Halomonas bluephagenesis* (*H. bluephagenesis*)*,* a novel halophilic chassis strain, can achieve continuous PHA synthesis under non-sterile conditions at high salt concentrations [72]. PHA [73] and HA [74] were both produced using in engineered *C. glutamicum*, an organism free of exotoxins and endotoxins. PLA also can be produced by engineered bacteria [75], which has led to its extensive medical use.

Moreover, *Chlamydomonas reinhardtii* (*C. reinhardtii*) is a new single-celled green alga with a short growth cycle, which often exhibits more stable protein expression for AMPs [71]. Most cellulose has been produced in *Acetobacteraceae*, *Gluconacetobacter* and *Komagataeibacter* [76]. In recent years, biosynthetic silk protein has been obtained using several model organisms such as *E. coli* [77], *B. subtilis* [78], *S. cerevisiae* [79] and Chinese hamster ovary cells [80].

#### Synthetic biology tools for BNBMs

Synthetic biology is a novel interdisciplinary field involving mathematics and systems biology that has been utilized to design new biological parts and biosystems to improve the properties of microorganisms and their products [71]. Various bio-tools, such as clustered regularly interspaced short palindromic repeats (CRISPR)—CRISPR associated protein 9 (CRISPR-Cas9), optimization of ribosomal binding sites and novel genetic elements have been introduced in recent years. These bio-tools can promote the biosynthesis of various natural biomaterials, reduce the cost, and adjust their physical properties.

The CRISPR-Cas9 tools, especially the CRISPRi systems, have been used successfully to control the biosynthetic flux of the PHA pathway and adjust the molecular weights and composition of the products. By simultaneously manipulating multiple genes in *E. coli* via CRISPRi, expressions of the *prpC* gene encoding 2-methylcitrate synthase can be changed to regulate the 3-hydroxyvalerate content in P(3-hydroxybutyrate-co-3-hydroxyvalerate) (PHBV) copolymers from less than 1 to 13% [81] and generate P34HB with 1.4 to 18.4 mol% of 4HB [82]*.* Similarly, the molecular weight of HA was increased by decreasing the expression of FtsZ (initiating cell division) and changing the distribution of cardiolipin in the membrane of *Bacillus subtilis* [83]. The CRISPR-Cas9-initiated fixed-point strategy was employed to successfully incorporate spider silk protein genes into the *Bombyx mori* genome, resulting in fibers that are as strong as native spider silk, with a high tensile strength of 1.2 GPa [84].

Using the CRISPR-Cas9 system, the function of endogenous genes was disrupted in BM-N cells with a mutation frequency of 30 – 40%. Similarly, zinc-finger nucleases have been used to insert the human lysozyme gene into the bovine β-casein locus, leading to human lysozyme knock-in in approximately 1% of the treated bovine fetal fibroblasts. The milk secreted by the resulting transgenic cows could kill Staphylococcus aureus (*S. aureus*) [85]. The cell-free biosynthesis system is an efficient way to produce rare, easily degradable or toxic AMPs, such as cecropin P1 with 31 amino acid residues and human beta-defensin 2 (hBD-2) [86].

The optimization of ribosomal binding sites increased PHB accumulation to 92% of cell dry weight [87] and achieved a high HA yield of 8.3 g/L [88]. Novel T7-like RNA polymerase-promoter pairs were designed to overexpress the PHB operon and cell-elongation cassette (i.e., *minCD*) under isopropyl-β-D-thiogalactopyranoside induction, resulting in an increase of PHB accumulation and cell lengths in *H. bluephagenesis* [89]. Furthermore, recombinant collagens (rCols) produced using synthetic biology techniques have attracted increasing attention, since the structure of collagen can be modified with functional protein fragments [90].

#### Metabolic engineering for BNBMs

Metabolic engineering is also a relatively new and interdisciplinary field that draws from metabolic flux optimization, outer membrane permeabilization, cell morphology engineering, and chromosomal integration to manipulate microorganisms to more efficiently produce natural biomaterials at low costs.

PHA-accumulation can be increased in engineered microorganisms via oxygen limitation with alcohol dehydrogenase (PadhE) [91], prevention of succinate semialdehyde loss by *gabD*-knocking out [92], intercepting the 2-methyl-malonyl-CoA to 2-methylcitrate cycle (MCC) [93, 94], overexpression of NADH (or NADPH) synthesis enzymes (e.g., UdhA) [92, 93], weakening the beta-oxidation cycle by *fadA B*-knockout [92], chromosomal integration of PHA synthetase [93, 95], inhibition of fission ring protein FtsZ or weakening of cell skeleton protein MreB [96], and OM-defective halophilic *H. bluephagenesis* strains with a low endotoxin content for better biosafety [97]. Similarly, a strong oxygen supply can both influence metabolic fluxes toward PHB and alginate synthesis in *Azotobacter vinelandii* (*A. vinelandii*) cells due to alginate acetylation [98]. Optimized carbon/nitrogen molar ratios in both the batch and feeding media for *E. coli* BL21 resulted in the highest dry-cell density (67.2 g/L dry cell weight) and human-like collagen production (10.8 g/L) [99]. Notably, BC from the glycerol medium showed the highest tensile strength at 83.5 MPa, with thinner fibers and lower porosity, compared with BC from glucose and fructose medium. These findings illustrate that some biomaterial properties can be regulated using different carbon sources [100].

Furthermore, due to weak promoter substitution or plasmid overexpression, *DivIVA* and *FtsZ* genes were regulated for HA synthesis in *C. glutamicum*, which generated different shapes including small-ellipsoid-like (DivIVA-reduced), bulb-like (DivIVA-enhanced), long-rod (FtsZ-reduced) and dumbbell-like (FtsZ-enhanced) cells [101].

#### AI and ML

A variety of prediction algorithms based on AI and ML can be established to analyze and learn the patterns of natural biomaterials, after which the algorithm can be used to find relevant characteristics and rules. Eventually, a combination of AI and ML may lead to the prediction and accurate design of completely novel, previously unknown functional biomaterials with a variety of TE applications. The N-terminal residues of known AMPs were used to predict new AMPs via neural network, quantitative matrices and support vector machine learning, which showed accuracies of 88.17%, 90.37% and 92.11%, respectively [102]. Using quantitative structural-activity relationships, initial high-throughput measurements of over 1400 random peptides in artificial neural network models were conducted and subsequently used to screen an in-silico library of approximately 100,000 peptides [103].

Another future utilization of AI may be to predict important factors for metabolic engineering, such as expression of multiple genes in a pathway [104], plasmid architecture [105], as well as the simplification of protein engineering [106] and pathway design [107], which improves the accuracy and fidelity of target product synthesis [108].

As AI and ML technology advances, the characteristics of biomaterials blends, composites, or novel materials could be predicted and categorized by AI and ML [109–111]. For example, ML predicted the glass transition temperature of PHA-based polymeric materials and the location of PHA-accumulating bacteria in a mixed microbial culture. Similarly, the tensile strength of PLA fused deposition models was accurately estimated by an artificial neural network [112]. Enzymatic kinetics of cellulose hydrolysis in a heterogeneous system have been described by an artificial network and compared using response surface methodology [113] (Table 1)

**Table 1:** Synthesis strategy, methods and results of natural biomaterials based on novel techniques and tools

Strategies	Natural biomaterials	Methods, the involved strains or cells, and results	References
Novel chassis strains/cells	γ-PGA	Produced in engineering bacteria for high production	[66]
	AMPs	Produced in *C. reinhardtii* for stable expression of AMPs	[71]
	PLA & PLGA	Produced in engineered *E. coli*	[72]
	PHAs	Produced in *H. bluephagenesis* under high salt concentration and non-sterile conditions	[72]
	PHAs	Produced in engineered *C. glutamicum* for PHAs with free exotoxins and endotoxins	[73]
	HA	Produced in engineered *C. glutamicum* for HA with free exotoxins and endotoxins	[73]
	Alginate	Produced in engineering bacteria for high production	[96]
	Cellulose	Produced in *Acetobacteraceae*, *Gluconacetobacter* and *Komagataeibacter* for a large proportion of cellulose	[76]
	Collagen	Produced in engineering bacteria for high production	[99]
	Fibroin	Produced in engineered *B. subtilis*, *S. cerevisiae* and Chinese hamster ovary cells	[78–80]
Synthetic biology tools	PHAs	Manipulated the PHA synthesis-related genes via CRISPR-Cas9	[94]
		Increased the production of PHB via optimization of ribosomal binding site	[87]
	HA	Increased the production of HA via optimization of ribosomal binding site	[88]
	Fibroin	Incorporated spider silk protein genes into the Bombyx mori genome via CRISPR-Cas9	[84]
	AMPs	Inserted *hLYZ* gene into the bovine β-casein locus via zinc finger nucleases	[85]
	AMPs	Produced cecropin P1 with 31 amino acid residues and hBD-2 in cell-free biosynthesis system	[86]
Metabolic engineering	PHAs	Increased the production of PHA via PadhE, GabD, MCC, UdhA, FadA, FadB, FtsZ and MreB	[91–94, 96]
	HA	Regulated HA via genes *DivIVA* and *FtsZ* down or up	[101]
	Collagen	Increased the production of human-like collagen via optimization of carbon/nitrogen molar ratios	[99]
	Alginate	Metabolic flux analysis shows higher production of alginate acetylation in the cultures with limited oxygen	[98]
	Cellulose	Biomaterial properties of bacterial cellulose can be regulated through metabolic flux	[100]
AI & ML	AMPs	Predicted new AMPs via neural network	[102]
	HA	Predicted multiple genes expression in a pathway of HA based on AI	[108]
	HA	Simplified pathway design of HA via AI	[108]
	PHAs	Predicted the Tg of PHAs via recurrent neural network	[108, 109]
	Cellulose	Predicted the location of PHA-accumulating bacteria in a mixed microbial culture by recurrent neural network	[111]
	Collagen	Deduced the tensile strength of PLA fused deposition models by Taguchi L9 orthogonal array	[108]
	Cellulose	Enzymatic kinetics of cellulose synthesis was described by artificial neural networks	[113]

*PLA* Polylactic acid, *PLG*A Poly (lactic-co-glycolic acid), *E. coli Escherichia coli*, *PHAs* Polyhydroxyalkanoate, *PHB* Polyhydroxybutyrate, *HA* Hyaluronic acid, *γ-PGA* Gamma-poly-glutamic acid, *AMPs* Antimicrobial peptides, *hLYZ* Human lysozyme, *hBD-2* Human beta-defensin 2, *Tg* Glass Transition temperature, *AI* Artificial intelligence, *ML* Machine learning, *H. bluephagenesis* Halomonas bluephagenesis, *C. glutamicum* Corynebacterium glutamicum, *CRISPR-Cas9* Clustered regularly interspaced short palindromic repeats—*CRISPR* associated protein 9, *adhE* Alcohol dehydrogenase, *GabD* Succinate-semialdehyde dehydrogenase, *MCC* 2-Methylcitrate cycle, *UdhA* Pyridine nucleotide transhydrogenase, *FadA* Acetyl-CoA C-acyltransferase, *FadB* Fatty acid oxidation complex subunit alpha, *FtsZ* Cell division protein, *MreB* Rod shape-determining protein

.

In addition, AI and ML have been used to help optimize the manufacturing of medical devices by optimizing numerous prilling process variables. AI and ML have been deployed in the fabrication of alginate core-shell beads of sunflower oil [114], PLGA microparticles produced by diverse microfluidic systems either in the form of single or multiple particles [115], as well as the classification and selection of fish gelatin packaging films produced with palm oil and plant essential oils [116].

## Rational design and preparation of BNBMs enabling with ECM featured properties

**Fig. 2:**
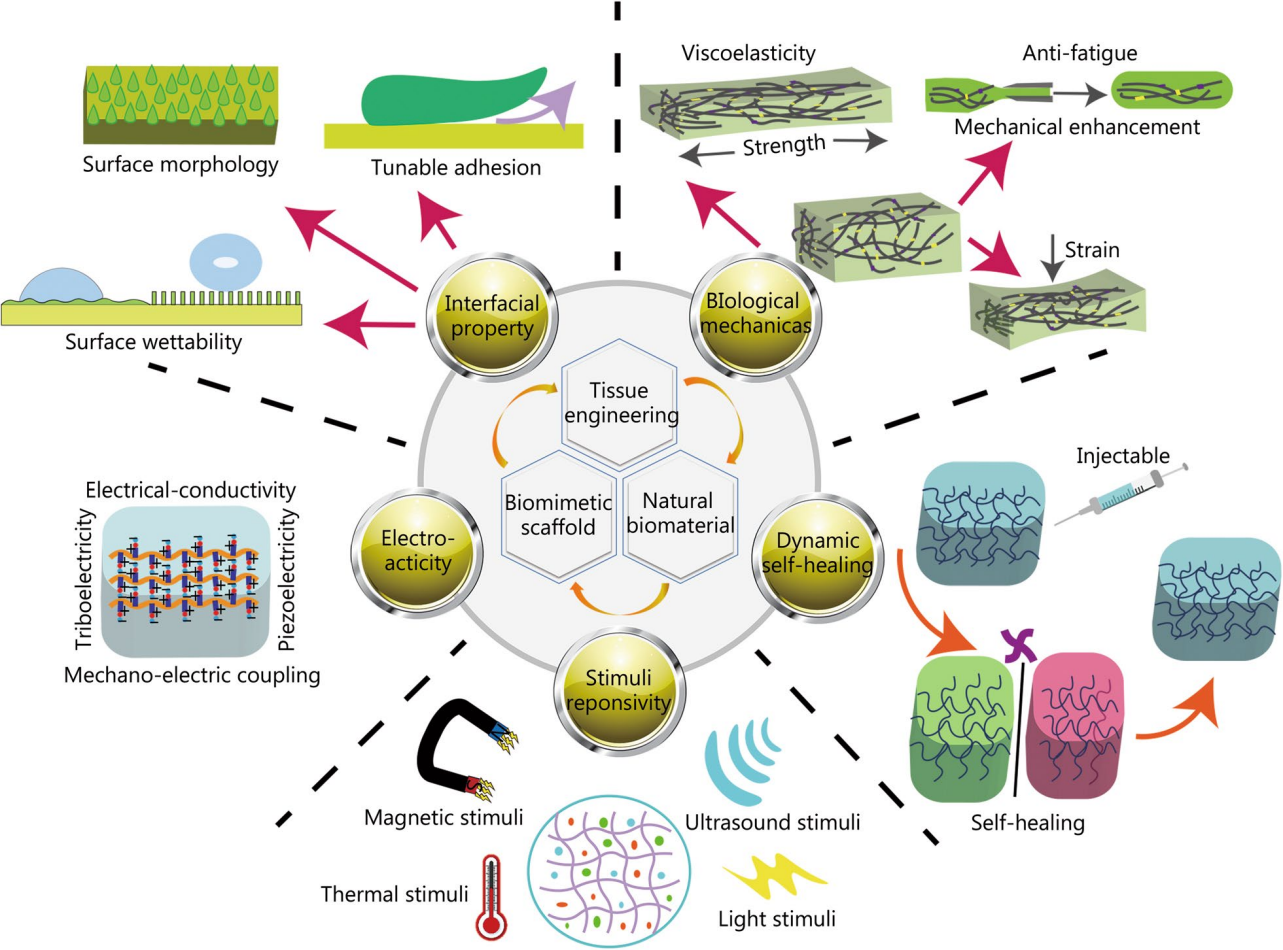
Schematically showing the rational design and preparation of biomimetic natural biomaterials (BNBMs) enabling with featured properties mimicking the tissue extracellular matrix (ECM)

Ideal TE scaffolds offer an ECM-mimetic environment for cell growth and tissue repair. Therefore, properties of the ECM, such as electro-conductivity, tissue-adaptive biomechanical properties, interfacial properties, stimulus responsiveness, mechano-electric coupling capacity, and so on, should be considered in the rational design process. As shown in Fig. 2, the development of BNBMs that mimic various biological and physicochemical characteristics of the natural ECM may have additional functionalities and enhanced therapeutic effects. Here, we summarized the design principles and preparation strategies of BNBMs with that mimic ECM features.

### Biomimetic design of electroactive BNBMs

Electroconductivity is one of the most essential features for normal electrophysiological activities and functions. As a consequence, to improve the electrical conductivity or electro-activity of BNBMs are crucial for mimicking the conductive coupling and electrostimulation of the native ECM [117]. The development of biocompatible and electroconductive BNBMs has been proposed to enhance the growth of electrical-active cardiac, neural, and musculoskeletal cells [117, 118]. One main challenge in matching scaffold conductance to that of local tissue is that most scaffolds use high-conductivity materials that are not biocompatible at high concentrations and can induce cell apoptosis or immune rejection [119]. Researchers have utilized conducting polymers (e.g., polyaniline and polypyrrole [120, 121]), nano-metallic wires, rods or dots [122], and carbon-based materials (such as carbon nanotubes [123], graphene [119]) to enhance the electrical conductivity of natural materials. Some of these materials have been successfully applied in experimental animal models and exhibited excellent biocompatibility. Very recently, Wang et al. [123] developed a novel conductive injectable hydrogel as functional cardiac patches to repair infarcted myocardium in rats and minipigs. These brand-new electroactive BNBMs based on elastin and carbon nanotubes are proposed to reconstruct electroconductive coupling, restore the electrophysiology of infarcted myocardium and promote cell proliferation [124]. The potential of BNBMs with enhanced electrical conductivity in TE and regenerative medicine is strengthened, and it is expected to enable better therapeutic performance for various bioapplications.

The mechano-electrical coupling (MEC) property is another electroactive feature by which an acute electrical feedback can be generated by the local mechanical environment and cellular electrophysiology through mechanosensitive subcellular components. Systems with MEC electroactive capacity can convert external mechanical energy into internal electric energy for the living organisms through a tissue-specific energy dissipation mechanism. Thus, the MEC property is an important functional characteristic for both biomechanical and biophysical behaviors of living systems. Enabling MEC properties in tissue scaffolds is of particular interest in organs necessitating biological MEC, such as the heart, bone, periodontium, and nervous tissues. In the case of cardiac tissue, normal heart rhythm requires the coordinated activity of numerous cardiac cells, with their rapid response and harmonious adaptation to changes in electrophysiological demands [124]. A key element in the turn-by-turn control and restoration of cardiac function is the restructuring of the electro-conductive coupling microenvironment and recovery of the electromechanical self-regulating mechanism [125].

Furthermore, the BNBMs with piezoelectric features can enable the integrated design of in vivo diagnostic and treatment tools, which opens a new field of research in BNBMs. Natural piezoelectric materials mainly include bone, wood, collagen, and spider silk [126–133], which not only have good biocompatibility, but also can be used for biosensing and biological power generation after modification [134–136], to achieve local motor/electrical coupling and maintain electrophysiological balance [137, 138]. Research has shown that many unmet healthcare needs, including biosensing, health monitoring, and repair of myocardial infarction, stand to benefit from expanding the fabrication and understanding of piezoelectric BNBMs.

In addition, the triboelectric effect, originating the bioelectricity through mechanical friction between two moving objects also endow the electroactive capacity of BNBMs [139, 140]. During the process of sliding, opposite charges are generated on the interface of the sliding materials. Charge accumulation and generation varies based on the composition and structure of the materials in motion, the surface area in contact, and the force applied during motion [141]. Researchers developed a series of composite triboelectric devices using natural biological materials with good cytocompatibility, such as cellulose [142], polyvinyl alcohol (PVA)/Ag [143], and spider silk/PVDF [144]. Moreover, since the charge generation phenomenon occurs mainly on the nanoscale, many triboelectric materials can be fabricated into various micro and nanoscale wearable devices to construct self-powered systems. Among these, the triboelectric nanogenerators are the most famous example of energy harvesting based on the triboelectric effect [141, 142]. These triboelectric materials and devices have enormous potential for human–machine interactions, health monitoring, peripheral nerve restoration, smart wearables and other medical applications [143, 144].

### Design of BNBMs with suitable biomechanics

Substantial studies over the past 20 years have established that the mechanical properties of the ECM affect cellular proliferation, growth, migration, differentiation and other behaviors. A successfully designed for BNBMs thus has to fit the mechanical requirements of the implant location and their cellular microenvironment. Through the intensive study of the mechanical characteristics of native ECM in different tissues, researchers gradually began to explore the biomimetic biomechanical design of BNBMs in terms of cytocompatibility, proliferation and tissue repair for potential applications in modern TE and regenerative medicine [145–147]. The poor mechanical performance of most original natural materials greatly restricts their application as scaffolds prospects. It is therefore of great significance to improve the biomechanical properties of BNBMs. To this end, Gong et al. [148] presented the construction of a double network hydrogel with high strength, offering a novel strategy for designing mechanically reinforced scaffolds. Strategies for improving mechanical strength based on an interpenetrating polymer network (IPN) or semi-IPN [149, 150], nanoreinforcement [151] and so on, were utilized to obtain scaffolds with the desired mechanical performances. Many studies have shown that nanocomposite reinforced BNBMs not only have an excellent toughening and reinforcing effect on polymeric bulk materials, but also bring additional benefits such as the inhibitory effect of nanomaterials on the generation of crazes and fatigue crack propagation [151]. Moreover, many biomimetic structural designs, such as honeycomb structures [152, 153], layer-by-layer stacking [154], and nacre-like mineralization [155, 156] have been developed to enhance the mechanical properties of scaffolds, which will be detailed later in "Biomimetic structural materials and their fabrication" Section.

Recent work has shown that the viscoelastic properties of the matrix can modulate these fundamental cellular processes and promote behaviors not observed in 2D and 3D in vitro cultured hydrogel systems [157]. These results indicate that BNBMs which match synthetic and natural ECM mechanics may improve the translational aspect of constructing tissue-specific in vitro cell or tissue models and TE applications. Generally, BNBMs with viscoelastic properties are based on biomimetic hydrogels with dynamic cross-linked networks containing multiplex covalent and non-covalent interactions, such as dynamic covalent acylhydrazone bonds, borate bonds, thioesters, guest-host complexes or electrostatic interaction [158–163]. For instance, a viscoelastic HA-based hydrogel can be formed with hydrazone covalent bonds as well as weak guest-host or hydrogen bond cross-links [162, 163]. With the introduction of weak noncovalent bonds into these covalent cross-linking networks, the viscoelasticity can be tuned independently by tuning the molecular weight of the polymeric constituents, the affinity of non-covalent interactions, the ratio between non-covalent and covalent bonds, and coupling of inert molecules to the constituent polymers as spacers [162–165].

In some TE applications, such as the regeneration of heart valves, bones and teeth, fatigue resistance is crucial for reliable and long-term replacement. Hence, TE scaffolds for such anatomies require fatigue resistant materials. Early on, researchers studied the underlying anti-fatigue mechanism of hydrogel and elastomeric systems and demonstrated that when the number of polymer chain entanglements exceeds the cross-linking point, the material’s toughness increases and the material exhibits anti-fatigue properties [166, 167]. The fatigue resistance of soft materials can be effectively enhanced through the introduction of additional structures, such as crystals [167], composites [168], and ordered folding units [169], into the original cross-linking network structure. Hydrogels with hierarchical structures were proven to exhibit higher fatigue resistance due to the synergistic effects between different scales. The biomimetic design and mechanism study for BNBMs with fatigue resistance enhanced our understanding of the fatigue failure behavior of biological tissues with complex hierarchical structures. With the burgeoning development of advanced material preparation and structural design strategies, various strategies for tough and fatigue-resistant BNBMs based on hierarchical microstructures are presented as promising for tissue-specific applications.

In addition, the injectability of BNBMs based mechanical responsiveness (such as shear thinning, stress relaxation, creep, relaxation, etc.) offers convenience and accessibility for in situ moldability, minimally invasive surgery, drug delivery, precise tissue targeting, and uniform material distribution. Therefore, injectable hydrogels are popular and widely used in biomedical applications such as TE and drug delivery. In recent years, in situ cell injection therapies are gradually being used in clinical settings, in which BNBMs play a pivotal role as a protective niche for cells. A variety of methods focusing on chemical [170–172], physical [173, 174], and multiple synthetic strategies [175, 176] were presented for preparing injectable hydrogels. Physically cross-linked injectable hydrogels rely on the involvement of tenuous intermolecular associations and reversible non-covalent bonds, which are in response to an environmental condition such as temperature, pH, ionic strength, or the existence of opposite ions during network configuration. By contrast, chemically cross-linked injectable hydrogels can in situ gel through forming irreversible covalent polymer networks from a soluble precursor using cross-linking agents or enzymes. The rapid and in situ-forming injectable hydrogels also facilitate the encapsulation and controlled release of living cells and bioinks and endow the capacity to match irregular defects.

### Biomimetic interfacial engineering of BNBMs

A precise host tissue-biomaterial interface is capable of regulating the cell-biomaterial interactions and the desirable performance of biomaterials in biological environment. Surface and interface engineering have been widely studied to tailor desired behaviors and functionalities into biomaterials to achieve better clinical performance. Thus, the interfacial engineering approaches as well as critical factors that determine tissue-specific functionality for BNBMs should be considered.

Surface wettability is the primary factor for dominating the protein adsorption, cell adhesion and other subsequent behaviors due to the essence of the dynamic liquid cell environment [177]. Superwettable (superhydrophobic, superhydrophilic and superslippery) surfaces are the most representative biomimetic examples for biological surfaces and interfaces to regulate the biomolecule and cellular adhesion behaviors [177]. The wettability of a material’s surface is determined by its surface morphology (surface roughness, microstructure) [178, 179] and inherent properties of the material (surface energy) [179]. Tuning methods of surface wettability are mainly divided into physical methods (such as plasma treatment, templating, spraying), chemical methods (electrochemical, self-assembly) and combined physicochemical methods (vapor deposition, etching). For instance, the introduction of an anisotropic striped surface pattern [178] and photo-induced gradient cross-linking layers enabled tunable surface wettability and creation of gradient soluble factors, cells, and microspheres in 3D hydrogels [179].

Moreover, to tailor the surface adhesion or anti-adhesion to a cell is one of the essential requirements for specific-propose tissue repair. For resisting undesirable adhesion and preventing adverse tissue-synechia, current anti-adhesion strategies mainly focus on pharmaceutical treatment and physical barriers or functional implant coating with the capability of inhibiting fibroblasts and anti-inflammation [180]. Contrary to antiadhesive materials, it may be favorable in many cases to form stable and firm wet tissue adhesion or even mucoadhesion performances. Wet adhesion presents a challenging task because when wet tissual interfaces interact, water separates the biomolecules on both surfaces and prevents tissue-material interactions, resulting in sharp decrease of tissue-material adhesion. In order to maximize the clinical utility, various biomimetic approaches have been adopted to enhance or optimize the integrin-mediated cellular interactions and adhesion, including incorporation or surface modification with bioactive molecules or bioadhesive ECM motifs, and macro- or nano-scale patterning of the biomaterials [181].

Besides the above-mentioned benefit on biomechanical properties, the topographical cues, such as surface microstructure, on TE scaffolds has significant influence increasingly become a focus of studies in recent years. Some technical approaches such as 3D printing [182], photolithography [183], plasma spraying [184], ion implantation [185], etc., have also been widely used for preparing hierarchical microstructured BNBMs. Scaffold-tissue interactions are critical for guiding and modulating favorable cellular and structural outcomes. Therefore, the surface microstructures of scaffolds are often designed and constructed aiming to improve biomechanical properties and bioactivity at the cellular and tissual level [186, 187]. Inspired by native wrinkle microstructures, Hou et al. [188, 189] developed a series of dynamic surface wrinkle microstructures, and systematically investigated the formation principles to assess the potential for wider biological applications of dynamic microstructures.

In addition, there are a large number of biointerfaces abundant in biological systems, for example, bone-to-connective tissues (ligaments, tendons, and cartilages) interfaces, which are responsible for transferring loads between tissues with significantly discrepant properties and functions. To modulate and biomimetic construct such natural tissue interfaces with unique microstructural properties and characteristics is beneficial for repairing gradient-connection tissues and achieving perfect therapeutic performance between tissues [190].

### Tunable properties of BNBMs based on stimulus responsiveness, self-healing, or injectable properties

BNBMs capable of self-healing and responding to external stimuli (including acoustic [191], magnetic [192], light [179], pH [193], thermal [194] stimulation) make them possible to recapitulate the ECM-mimetic dynamic environment ex vivo and achieve controllable manipulation of physical and biochemical cues to the cell and tissue. To this end, researchers have presented a series of stimulus-responsive technologies and material systems (e.g., hydrogels with cells, growth factors, microspheres, etc.) for TE applications. For instance, magnetically responsive BNBMs prepared by introducing magnetic nanoparticles (MNPs) into hydrogel networks are advantageous in biological applications considering their rapid magnetic response, precise spatiotemporal control, and non-invasive remote operation [195]. The preparation methods of magnetically responsive hydrogels mainly include the following: 1) MNPs are mixed with the hydrogel precursor solution [192]; 2) MNPs are co-precipitated in the prefabricated hydrogel matrix [196]; and 3) MNPs are used as hydrogels cross-linking agents [197]. In addition, a magnetic patterning approach has been applied to construct complex tissue-engineered articular cartilage with a depth-dependent cellular structure similar to native cartilage [198].

The sound sensitivity of BNBMs opens a novel avenue for sonodynamic therapies. This effective acoustic-based method combining the low-intensity ultrasound and drugs (acoustic sensitizers) has been recognized as a promising approach for drug delivery and scaffold morphology modulation. Researchers mainly combine sonosensitizers (such as porphyrin analogs) with natural biomaterials to prepare sound-responsive scaffolds and apply them to TE and regenerative medicine [191]. Some acoustically responsive scaffolds were utilized to achieve sequential delivery of different growth factors encapsulated in a perfluorocarbon emulsion by simply controlling the acoustic parameters [199].

Generally, pH, as one of the most basic factors and indicators for normal cellular and tissue microenvironments, may reflect a considerable variation of physiological status. Accordingly, BNBMs endowed with pH responsivity are capable of releasing bioactive cargos in response to physiological changes and thus enhanced function as tissue scaffolds. Many ionizable groups or acid-cleavable bonds are responsive to environmental pH changes, such as Schiff bases [193], hydrazone [200], acetal [201], and β-thiopropionate [202]. For example, Gan et al. [203] reported a pH-responsive surface-functionalized mesoporous silica nanoparticle system for simultaneously delivering both growth factor bone morphogenetic protein-2 and the drug dexamethasone for osseointegration.

Furthermore, the thermal-responsive BNBMs has received significant attention due to their controllable injection and drug release [204–207]. Most researchers have been focusing on the development of efficient and rapidly responsive thermosensitive biomaterials such as Pluronic F127 in a non-covalent manner, sodium chitosan/β-glycerophosphate, and furan dimer gelatin covalently cross-linked by click chemistry with maleimide-functionalized polyethylene glycol (PEG) [204]. It can be foreseen that these temperature-sensitive BNBMs have great promise for clinical therapeutic applications such as responsive on-demand antibiotic release to combat bacterial infection in wound dressings.

Researchers also focused on redox-responsive BNBMs to regulate excess reactive oxygen species or glutathione (GSH) in the human body, achieve redox balance, repair tissue damage, and kill tumor cells [208–210]. Kim et al. [209] prepared epidermal growth factor-loaded cysteamine-cross-linked BNBM scaffolds, utilizing cysteine to respond to GSH to achieve targeted release of bio-factor and enhance the desired therapeutic effect. The construction of this BNBM-based redox response system has broad prospects in the fields of tissue repair and cancer therapy.

Besides, many other factors can be also considered in the responsive design of BNBMs, such as enzymes, light, etc. For the former, growth factors can be released by modifying matrix metalloproteinases in BNBMs [210], or BNBMs can be prepared as nanozymes to release related factors through intracellular cascade reactions, thereby achieving tissue repair. For photoresponsiveness, designs such as photodynamic therapy and infrared-to-ultraviolet light conversion can be utilized for medical applications such as therapy and diagnosis.

Considering that the applications of BNBMs are diversified and often susceptible to mechanical disruption, the requirements for BNBMs in tissue-specific performance and challenges in various harsh physiological conditions have also increased. Inspired by the self-healing properties of human tissue after minor damage, self-healing BNBMs were designed and developed mainly based on non-covalent interactions (such as hydrogen bonds, van der Waals forces), weak covalent bonds (for instance, acylhydrazone bond), ionic complexes and other design methods [25, 41, 56, 179]. The self-healing properties endow BNBMs capable of catering to the clinical requirements and promising to replace the traditional brittle stents in the fields of orthopedics, heart, skin, and muscle repair [25, 41, 179].

## Biomimetic structural materials and their fabrication

**Fig. 3:**
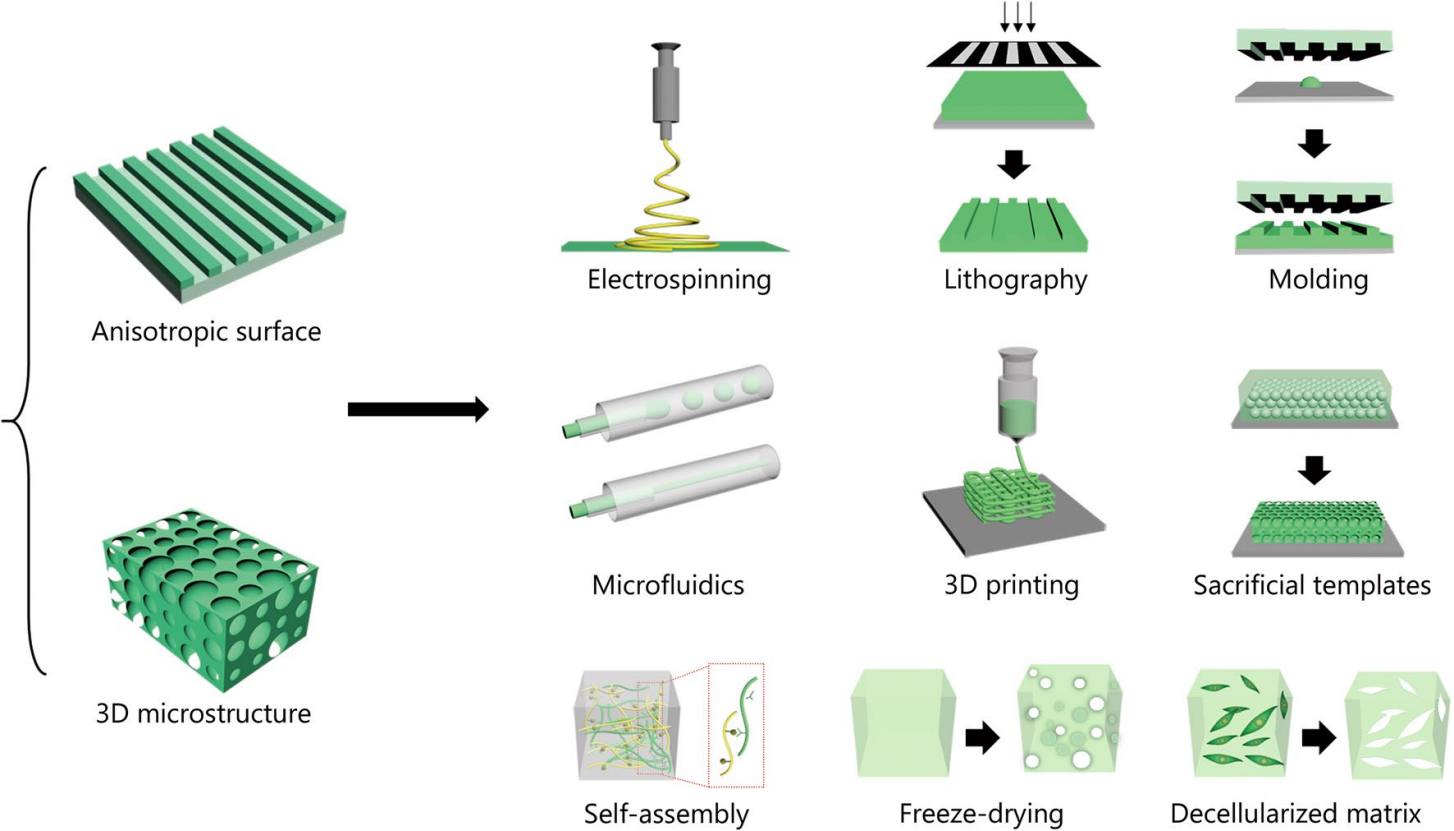
Typical topologies and fabrication strategies of the structural biomaterials

Microstructural properties should be considered when designing biomaterials for TE and regeneration because microstructure not only determines the mechanical properties of the biomaterials but also affects their interactions with cells and tissues [211–213]. When the materials are used in a damaged area, it is favorable to have a microstructure similar to that of the tissues to facilitate cell migration and infiltration. Additionally, bioactive molecules, such as drugs, growth factors and so on, can be integrated into the microstructure for synergistic therapeutic effects. Here, we summarized the typical topologies and fabrication strategies of structural biomaterials used in tissue regeneration, as shown in Fig. 3.

### Biomimetic structures in tissue regeneration

#### Anisotropic surface

The surface of the biomaterial is in direct contact with the damaged area. Therefore, the surface features, especially the topology of the biomaterial, are critical in TE applications, especially in facilitating cell alignment and polarization. Anisotropic surfaces, such as aligned fibers, structural patterns, or oriented materials, enable the use of BNBMs as a tissue scaffold that leads to an orientation-dependent mechanical response. To date, many kinds of microstructures have been developed, including grooves, ridges, caves, convex patterns, and so on.

Grooves and ridges are the most commonly used anisotropic topography, and they can modulate cell polarization significantly. It is worth noting that although they can induce cell polarization at different scales, the mechanisms are different [178, 214]. When these groove and ridge patterns are used at the micro-scale, they provide spatial limitation to the cells and activate the formation of pseudopodia. However, when they are used at the nanoscale, they regulate the orientation of adhesomes by the myosin-based intracellular force. The depth of the grooves is another parameter that affects cell behavior. For example, deeper grooves caused increased differentiation, alignment and neurite elongation of murine neural progenitor cells [215].

Concavity is another typical micropattern for guiding cell behaviors. Chen et al. [216] cultured human osteosarcoma MG-63 cells on the substrates with concave patterns of different sizes. It was found that larger concaves patterns enhanced the proliferation of cultured cells because the substrate was suitable for cell attachment, while the smaller concave patterns promoted osteogenic differentiation due to the strong topological stimulation. Similarly, convex topology can improve the wettability and surface energy of the substrate, leading to enhanced capability in promoting cell adhesion, proliferation, differentiation, and achieved the best effect when the micropattern size is close to the size of cells [217].

#### 3D microstructures

Although many advances have been made in patterning the microstructure of scaffolds, many of these advances have been in planar scaffolds [190, 194], which may not be suitable for the repair of volumetric defects. The absence of 3D microstructure within the biomaterial may limit the vascularization of the regenerated tissues and decrease the repair efficacy. Conversely, BNBMs with 3D microstructure allow cell infiltration as well as mass transfer, supporting vascularization and ECM deposition. It should be noted that the mechanical properties are also closely related to the pore interconnectivity. Therefore, it is crucial to find a balance between meeting the demands of mechanical properties without sacrificing advantageous biological effects. For example, researchers developed a series of porous microfluidic hydrogel particles based on BNBMs for tissue repair [218–220]. These biomaterials had porous structures for the loading and release of drugs, as well as inducing the proliferation and differentiation of cells. Dai et al. [221] designed a tissue scaffold using methacrylated hyaluronic acid for bone/cartilage tissue regeneration. The HA-based scaffold had radially oriented pores, which were used to recruit and orient cells. When the scaffolds were implanted at sites of cartilage defects in experimental animals, favorable cartilaginous regeneration was achieved.

Nanofiber scaffolds enable superior control and generation of microstructures. By tuning the parameters of electrospinning, scaffolds with controllable nano- and micro-structures can be readily prepared [222–224]. Rnjak-Kovacina et al. [225] presented an electrospun fibrous elastin-collagen composite scaffold for the regeneration of skin. The obtained materials not only maintained inherent bioactivity, but also had tunable microstructures. The synergistic effect of the natural materials and the optimal microstructure resulted in favorable cell attachment and proliferation.

Inverse opal scaffolds are biomaterials with ordered 3D porous microstructures [226–229]. The size of the interconnected pores is homogeneous and controllable, which results in the efficient transfer of nutrients and wastes, leading to a uniform distribution of cells. Additionally, the uniform pores make it possible to evaluate effects on cell infiltration [230]. It is also feasible to functionalize inverse opal scaffolds with drugs or signal molecules to enhance their therapeutic effects. For example, Osathanon et al. [231] prepared an inverse opal fibrin scaffold modified with alkaline phosphatase, which can promote bone formation, whereby significant mineral deposition and bone regeneration was observed on the scaffold.

### Fabrication of biomimetic structural materials

#### Electrospinning

Electrospinning is a common technique that can generate polymer fibers with a desired size [232, 233]. There are three main components of electrospinning systems, including a high voltage power source that provides the electrostatic field, a material delivery system that delivers the material continuously, and a substrate that collects the generated fibers. When the system is running, polymer solution is extruded and forms a Taylor cone under an electric field. During spinning, the solvent is evaporated, and the jet is solidified. By adjusting the electrospinning parameters, including the solution’s viscosity, conductivity, and surface tension, as well as the electrostatic field, temperature, and humidity, the diameters of the fibers can be tuned from the nanoscale to microscale [234].

However, it should be noted that many solvents used for electrospinning are halogenated compounds, which have high toxicity and negative effects on the environment. To solve this problem, more biocompatible “green solvents” have been developed. For example, Zhou et al. [235] reported a collagen/hydroxyapatite (HAP) composite fiber with an excellent microstructure fabricated by electrospinning. Rather than using potentially cytotoxic organic solvents, they dissolved type I collagen in a solution of phosphate buffered saline (PBS)/ethanol. The solution was desalinated and co-electrospun with HAP to form collagen/HAP composite fibers, which exhibited not only better mechanical properties, but also had a microstructure similar to natural bone.

#### Lithography and molding

Lithography is a widely used technology capable of precisely fabricating intricate biomaterials. Like electrospinning, lithography is flexible in operation and relatively inexpensive. Photolithography and soft lithography are the most common lithography methods for preparing BNBMs [141, 183]. Photolithography uses ultraviolet (UV) irradiation to cure the photoresist layer through a photomask [236]. This strategy can produce materials with high throughput and thus, the cost of each patterned element is low [188, 237, 238]. However, several inherent weaknesses such as the wavelength and diffraction effect of the light may limit their application in developing microstructures with ultrahigh precision down to the nanometer scale. By contrast, soft lithography employs patterned molding or stamps with high resolution to prepare biomaterials with pre-designed patterns [239]. This method is simple, fast, and low-cost, while avoiding residues of initiators and free radicals which may remain in photolithography processes. These features allow it to be employed for constructing a variety of structural biomaterials. Moffa et al. [240] presented a micro-grooved scaffold with oriented fibers for engineering vessels. In this work, they prepared a patterned polydimethylsiloxane mold and used it as a template to form a microgrooved gelatin. Next, electrospinning technology was used to fabricate nanofibers on the gel in order to provide nanoscale cues. By tuning the collection parameters, the micro- and nano-scale topographic cues could be aligned in random, parallel, or perpendicular relative directions.

#### Microfluidics

Microfluidics is a technology that can precisely modulate fluids on the sub-millimeter scale [115, 241–245]. Because the dimensions of the fluids manipulated by microfluidics are at the microscale, their specific surface area increases and the fluids exhibit different behaviors from those of drops on a macroscopic scale [245]. Additionally, the microfluidic systems can integrate many channels, so that various fluid phases can simultaneously flow in the systems. Based on the diverse interactions among the fluid phases, various kinds of fibers and droplets can be generated [115, 224, 244]. Fibers are fabricated in microfluidic systems based on the concentric flow of a core and a sheath fluid via two channels, while droplets are generated by the action of liquid-liquid interfacial tension and shear force [245]. The biocompatibility of the fibers and droplets is determined by the fluids used in the fabrication process and their interactions with microfluidic chips. Surfactants are often used in the fabrication process to stabilize the fluid and the generated products, but they may have negative effects on cells. In some cases, photopolymerization is combined with microfluidic technologies, which may leave residual photoinitiators and free radicals. Therefore, the fibers and droplets prepared using these systems should be washed carefully before use.

Thanks to the ability to control fiber or droplet size during fabrication, microfluidic technology has tremendous potential for preparing BNBM-based functional scaffolds. For example, Yu et al. [246] developed a metal-organic framework (MOF)-loaded microfiber using microfluidics. In their work, various solutions were pumped into a coaxial capillary microfluidic chip and formed shell-ore microstructures via interactions between Ca^2+^ and sodium alginate, as well as nicotinic acid and Cu^2+^ or Zn^2+^ solutions. In a different approach, Yang et al. [247] integrated microfluidics with electrospray technology to fabricate stem cell-laden alginate hydrogel capsules. The fluids at the end of the microfluidic chip were cut into droplets composed of alginate shells and stem cell cores by the shear force of an electrostatic field. The shells gelled immediately when the droplets were collected in the calcium chloride solution. This unique core–shell structure provided a good microenvironment for cell culture and the following in vivo applications.

#### 3D bioprinting

3D bioprinting is a novel technology for preparing biomaterials and is generally defined as the application of additive manufacturing in biomedical fields [38, 182, 248]. Biomaterial models are designed by computers before fabrication, while microfluidics and photopolymerization are often employed to manipulate the bioink to fabricate designed biomaterials layer-by-layer. Therefore, the biocompatibility of the prepared materials is mainly determined by the employed bioinks. According to the fabrication methods, 3D bioprinting can be classified into four categories of extrusion, inkjet printing, laser-induced forward transfer, and vat polymerization.

Since 3D bioprinting can prepare biomaterials with a programmed microstructure, it has been widely utilized to develop biodegradable scaffolds with suitable microstructure and spatial cell distribution [249]. Based on this strategy, Wang et al. [249] realized a living photosynthetic scaffold composed of microalgae, alginate, and gelatin methacrylate (GelMA) similar to natural tissues or organs using an extrusion strategy. They used gelatin fluid containing calcium chloride and the mixture solution of microalgae, alginate, and GelMA as the inner phase and outer phase, respectively. The cross-linking between Ca^2+^ and alginate formed the hollow fibers which were subsequently irradiated under UV light to photopolymerize the GelMA component. Furthermore, it was demonstrated that the resultant hydrogel fibers could be stacked in situ on tissue and form a 3D scaffold, indicating that this method could be a novel 3D bioprinting technology. Boland et al. [250] employed inkjet technology to prepare cell multilayers with 3D structures. They fixed a platform on an elevator stage which lowered after each printed layer and thus, the cross-linking could be continued until the elevator stage reached the bottom. By using this system, they acquired the 3D construct with the desired form and microstructure for cell culture. Vat polymerization is a 3D printing method which solidifies liquid ink in a layer-by-layer manner using photopolymerization, which can rapidly generate structural materials with high-resolution architectures. Due to these features, it has appealed to many researchers. For example, Wang et al. [251] employed this method to cross-link a bioink made of GelMA containing eosin Y photoinitiator under visible light irradiation. The formed hydrogel exhibited the designed shape and micropattern, while cells could adhere and proliferate well inside the hydrogel.

#### Sacrificial template

The fabrication of materials with a specific microstructure often requires machining technologies with high precision. Many of these technologies are difficult to operate at scale and are expensive. By contrast using materials that possess unique micro- or nano-structures as sacrificial templates to fabricate the biomaterials is an easy and feasible strategy [251, 252]. The pre-polymerization solution can infiltrate the small structure of the templates and form a polymer network after polymerization, after which suitable corrosives are employed to etch the templates. The resulting materials must be washed thoroughly before use for cell culture or in animals to remove the residual toxic reagents. For example, Zhang et al. [253] fabricated a hollow conduit with porous inner walls for drug loading and peripheral nerve regeneration. They used silica nanoparticles to form a thin film with periodic layers on the outside of a glass capillary. The thin silica film was used as template to obtain the natural protein-derived conduit by pre-gel infusion, UV irradiation, and sacrificial template etching. Kong et al. [254] combined the template with a photomask to prepare a hydrogel film for corneal stroma regeneration. In their work, the colloidal crystal film was infiltered with photopolymerizable natural polymer solution, and a photomask was added to the film prior to UV irradiation. After sacrificial template etching, the hydrogel film retained both the nanostructures inherited from the colloidal crystal array and the microscale grooves from the mask (Table 2).

**Table 2:** Design principles, preparation methods, and applications of biomimetic natural biomaterials

Modification	Biomaterials	Properties	Methods	Principles	Applications	References
Electroactive design	Collagen/chitosan	Conductivity	Loading with conductive substances or modification of electroactive functional groups	Electronic vacancy/movement, ionizable groups	Cardiac/skin/nerve/muscle tissue engineering, diagnosis	[117–125]
	Collagen/PVDF/M13-bacteriophage	Piezoelectric	Loading with piezoelectric materials	Ordered nature of nano- or liquid-crystalline in biomaterials	Biosensing monitoring, health monitoring, dentistry, cardiac/skin tissue engineering	[126–138]
	Cellulose/silk	Triboelectric	Loading with triboelectric materials	Charges generated by friction	Human–machine interactions, health monitoring, peripheral nerve restoration, smart wearables	[139–144]
Biomechanics design	Cellulose/collagen/alginate	Mechanical enhancement	Bionic structuring, multiple networks, doped nanomaterials	Nano enhancement, improving cross-linking density	Cartilage/bone/muscle tissue engineering	[148–156]
	HA/collagen/alginate	Viscoelasticity	Chemical modification, mimics ECM dynamic mechanics	Abundant dynamic covalent bonds	Cell niche, mediating cell behavior, ophthalmology, drug delivery, tissue regeneration	[157–165]
	PVA/HA	Anti-fatigue	Adding crystals composites and ordered folding units, introduction of hierarchical structure	Multiscale design, polymer chain entanglements	Cardiac/skin/neuro/cartilage/bone/muscle tissue engineering,	[166–169]
	CMCS/PEG/HA	Injection	Chemical/physical modification	Weak (non-covalent) cross-linking, shear thinning	Drug delivery, in situ moldability, targeted therapy	[170–176]
Interface design	PEG/gelatin	Superhydrophilicity or superhydrophobicity	Plasma treatment, template, spraying, electrochemical, self-assembly, vapor deposition, etching	Surface roughness, surface energy	Cell culture/gradient scaffold construction, skin repair, dentistry, artificial vascular	[177–179]
	Retinin	Anti-adhesion	Chemical modification, spraying,	Physical barriers, reduced contact area/surface energy	Abdominal wall defect treatment, anti-protein adsorption	[180]
	PEG/Chitosan	Wet adhesion	Chemical modification, electrochemical, etching	Electrostatic interaction, Strong water absorption	Wound dressing, preventing infection, wet tissue adhesion, wound closure hemostasis	[181]
Stimulus responsiveness design	PEG/PVA/MNP	Magnetic field response	Embedding magnetic nanomaterials	Moving charge or changing electric field	Cartilage/bone tissue engineering, diagnosis	[192, 195–198]
	Porphyrin/fibrin	Sound sensitivity	Embedding sonosensitizers nanomaterials	High acoustic sensitization activity	Controlled drug release, tissue engineering,	[191, 199]
	PEO/chitosan	pH sensitivity	Chemical modification	Ionizable groups or acid-cleavable bonds	Targeted drug delivery, tissue regeneration	[193, 200–203]
	Chitosan/gelatin	Temperature sensitivity	Adding thermosensitive polymers	Low glass transition temperature	Drug delivery, tissue regeneration, injection-based cell therapy	[204–207]
	GSH/mussel/DTT	Redox response	Chemical modification, physical doping	Redox-responsive chemical bonds, cascade response	Targeted drug delivery, cancer treatment	[208, 209]
Self-healing design	PAA/gelatin/SA	Rapid self-healing	Chemical modification, ion complexation	Weak sacrificial links	Skin/nerve/muscle/cartilage tissue repair	[179]
Microstructure design	HAP/HA/PLGA/collagen	Anisotropic surface	Electrospinning, lithography and molding, microfluidics, 3D printing, sacrificial templates, self-assembly, freeze-drying	Surface roughness, microscale effects, topology	Guiding cell fate, nerve/bone tissue regeneration	[214–231]
	Collagen/carrageenan	3D microstructure			Vascularization, drug release, transfer of nutrients and wastes, mediating cell fate, cartilage/skin/bone tissue engineering	[235–254]

*PVDF* Polyvinylidene difluoride, *ECM* Extracellular matrix, *PVA* Polyvinyl alcohol, *HA* Hyaluronic acid, *CMCS* Carboxymethyl chitosan, *PEG* Polyethylene glycol, *HAP* Hydroxyapatite, *PLGA* Poly (lactic-co-glycolic acid), *MNPs* Magnetic nanoparticles, *PEO* Polyethylene oxide, *GSH* Glutathione, *DTT* Dithiothreitol, *PAA* Polyacrylic acid, *SA* Sodium alginate

#### Bio-inspired self-assembly or self-organization

Self-assembly can be used to form stable structures through non-covalent interactions among molecules including hydrogen bonding, electrostatic force, and Van Der Waals force [190, 255–259]. For example, peptides with complementary chemical and spatial structures can self-assemble into fibers [260]. Furthermore, fibers can be organized to form hydrogels with specific nanopores [261]. These biomimetic self-organization strategies avoid toxic chemical reagents (such as chemical cross-linker), while the fibers and hydrogels degrade into nontoxic small molecules. Kumar et al. [262] used self-assembling peptides to form nanofibrous hydrogel scaffolds that facilitated angiogenesis. They employed a multi-domain vascular endothelial growth factor 165 (VEGF-165) mimicking peptide named SLanc. The scaffold could be dissolved in deionized water and rapidly form hydrogels when mixed with negatively charged multivalent ions.

There are several studies demonstrating that BNBMs can also be developed by the self-assembly of ECM secreted by cells. L’Heureux et al. [263] demonstrated that fibroblasts could form an ECM substrate with suitable mechanical strength by in vitro cell culture. Based on this cell culture system, they prepared a large quantity of cell-assembled ECM and explored its clinical applications, such as ECM yarns for surgical sutures.

#### Freeze-drying

Freeze-drying, also known as lyophilization, can be used to prepare porous biomaterials from polymeric solutions without additional cross-linkers and thus, can be employed for preparing materials with superb biocompatibility [264, 265]. The preparation process of freeze-drying includes three steps: dissolving polymers in water to acquire a polymer solution, freezing the solution in a container or a mold, and removing the ice from the frozen solution with a lyophilizer. When water is frozen, it acts as porogen. Accordingly, the porosity and pore size of the materials is dependent on the rate and method of freezing as well as the freeze-drying parameters. Rapid freezing hinders the formation of large ice crystals and results in small pores. Wang et al. [266] prepared a series of frozen scaffolds based on natural biomaterials for the repair of myocardial infarction. It is worth noting that a high vacuum is generally necessary to maintain pores during sublimation. If the vacuum is lost prior to the complete removal of ice, the scaffold may collapse on itself. Stokols et al. [267] explored the feasibility of freeze-drying agarose to form a scaffold with uniaxial linear pores for repairing spinal cord injury. Although the scaffold was not cross-linked with any chemical strategies, it remained stable under physiological conditions for a long time.

#### Decellularized matrices

Decellularization is a technique that removes all cells from a natural tissue via physical, chemical, or biological methods and leaves behind only the ECM of the tissues or organs [268]. The resulting scaffolds are also called decellularized ECM (dECM), and they may be favorable options for tissue regeneration since they retain the original tissue microstructure. Theoretically, recellularization of dECM scaffolds can recover the physiological functions of the decellularized organs due to their similarities to physiological composition and microstructure, which endows them with excellent biocompatibility after removal of the decellularizing solvents [269, 270]. For example, Ott et al. [269] perfused heart tissue with a mixed solution of heparinized PBS and adenosine followed by sodium dodecyl sulfate. After washing with deionized water, Triton-X100, and PBS containing antibiotics, the structurally functional, yet decellularized heart was obtained. Using a similar perfusion method, other organs, such as liver, have been successfully decellularized [270].

Interestingly, decellularized matrices can retain some of their physiological function without recellularization. Xu et al. [271] presented a liver-mimetic detoxication device using polydiacetylene nanoparticle-functionalized decellularized liver scaffolds. The decellularized scaffold provided 3D architecture for nanoparticle immobilization and blood perfusion and could remove pore-forming toxins from the blood.

Since decellularized tissues are derived from native tissues, they have many advantages over synthetics scaffolds [272, 273]. Since the components and microstructure of the ECM are dependent on their source organs, the physical structure and chemical composition of dECM from different tissues are distinct and arguably more suitable for specific applications. Singelyn et al. [274] dissolved decellularized porcine myocardial tissues using pepsin to form an injectable solution, which could form nanofibrous hydrogels when treated with heat. When the solution was injected into experimental animals, it formed a hydrogel capable of facilitating cell infiltration in vivo. In another study, Hong et al. [275] confirmed the capability of decellularized brain tissue hydrogel to promote the repair of spinal cord injury. Further studies of decellularized tissue hydrogels from dermal [276], bone [277], and blood vessels [278] indicate this strategy is suitable for regenerating various tissue types.

## Application of BNBMs for TE and regenerative medicine

### Applications of component-mimetic biomaterials

Collagen is the main component of ECM in all connective tissues in the body, and more than 27 types of collagen were found to play critical role in supporting mechanical stresses such as tendon, skin, cartilage, and bone tissues. Collagen-based scaffolds are enlightened to treat various tissue injuries. For example, Rho et al. [279] prepared collagen nanofiber membranes by electrospinning to promote the migration of keratinocytes at the early stage of wound healing. Compared with 2D collagen membranes, 3D collagen scaffolds are better candidates for skin regeneration. Ahn et al. [280] developed 3D porous collagen scaffolds with controlled pore sizes. The 3D scaffolds sustained uniform fibroblast dispersion and complete coverage with keratinocytes, similar to that of full thickness skin. Similarly, Ng et al. [281] applied the macromolecular crowding to 3D bioprinting and fabricated a 3D hierarchical porous collagen-based hydrogel scaffold, which could be a potential substrate for skin wound healing and skin TE.

Similar to skin, tendon and ligaments are also mainly composed of collagen fibers [281]. Tendinous and ligamentous collagen fibers are remarkably strong, having one of the highest tensile strengths among soft tissues in humans. However, reconstructed collagen fibers are usually too weak to support daily load bearing. To overcome the current shortcomings of tendon repair scaffolds, Francis et al. [282] developed a new electrospun tendon mimetic material consisting of aligned poly (D,L-lactide) (PDLLA) and type I collagen fibers. The addition of PDLLA significantly improved the mechanical properties of the synthetic scaffold. This study further confirmed the necessity of maintaining cell orientation, which resulted in the upregulation of tendon-related genes after three days of co-culture with human stem cells.

In addition to the inorganic HAP matrix, approximately 1/3 of bone is composed of an organic matrix, 90% of which is type-I collagen [5, 12]. In the clinic, commercial collagen sponges, porous HAP scaffolds and bioceramics, which can mimic the organic or inorganic component, are widely used as plugs to fill bone defects, particularly after orthopedic surgery [24]. In recent years, more hybrid scaffolds have been developed. For example, Inzana et al. [283] fabricated a 3D printed scaffold composed of collagen and calcium phosphate for bone repair. The 3D printed scaffolds were osteoconductive and the new bone growth rate matched the degradation rate of the scaffold in a murine femoral defect model for 9 weeks after implantation. In addition to 3D printing, biomineralization is another common method for the fabrication of bone TE scaffolds. Yu et al. [284] prepared a mineralized collagen-HAP scaffold which showed an improved osteogenic effect, resulting in enhanced bone regeneration. Mirkhalaf et al. [285] developed a series of novel bioceramics by doping different concentrations of magnesium and iron into Baghdadite (a Zr-Ca-Silicate: Ca_3_ZrSi_2_O_9_). The resulting 3D printed bioceramics were 2–5 times stronger than other bioceramic scaffolds, while retaining a similar porosity. Additionally, the bone-inducing activity of the Mg-doped Baghdadites is 2.2 times higher than that of normal Baghdadite [285]. However, 3D-printed bioceramic scaffolds require high-temperature sintering, which may affect the accuracy of the final volume and surface structure. Yang et al. [286] reported uniform 3D printed tricalcium silicate bioceramic scaffolds with controlled spatial structures that were stabilized at room temperature. The nanotopography was modified on the surface of the pore walls within the tricalcium silicate scaffolds, resulting in significantly enhanced bone mesenchymal stem cells attachment, growth and alkaline phosphatase activity in vitro.

The main components of cartilaginous ECM are proteoglycans (e.g., chondroitin sulfate and HA) and type II collagen. Artificial cartilage scaffolds should be able to influence the phenotype and cell behaviors of chondrocytes [287]. A variety of natural hydrogels with favorable characteristics such as high-water content, optimal biodegradation, adjustable porosity, and biocompatibility matching that of the natural cartilaginous ECM have been developed as some of the most suitable scaffolds for cartilage regeneration. Little et al. [288] utilized type II collagens, HA, and chondroitin sulfate to construct an ideal microenvironment that preserved the normal chondrocytes phenotype. One of the most noteworthy advantages of this scaffold was its ability to promote chondrocyte gene expression, leading to the secretion of type II collagen and proteoglycans. Similarly, Ko et al. [289] discovered that the expression of genes encoding aggrecan, type II collagen, and cartilage oligomeric matrix protein was significantly enhanced within the scaffold with the addition of chondroitin sulfate and HA. Moreover, the secretion and accumulation of proteoglycans on this scaffold were also markedly increased.

### Applications of structural-mimetic biomaterials

Physical stimulation provided by the structure (e.g., surface topology and 3D architecture) of regenerative scaffolds is capable of regulating cell behaviors, including the proliferation, migration, differentiation, expression of genes related to tissue repair, as well as the maintenance of stem cell phenotypes and pluripotency [290–292]. In the following sections, we summarize the structures of mimetic biomaterials and their application in regenerative medicine (Table 3).

**Table 3:** Representative commercial biomaterial products for tissue regeneration

Products	Company	Intended use
Apligraf	Organogenesis Inc	Standard therapeutic compression for treatment of non-infected partial and full-thickness skin ulcers
Dermagraft	Advanced Tissue Sciences, Inc	Treatment of full-thickness diabetic foot ulcers
Integra	Integra Life Sciences Corp	Partial thickness burn wounds
Dermagraft	Smith and Nephew Wound Management	Treatment of wounds related to recessive dystrophic epidermolysis bullosa
GraftJacket	Wright Medical Technology	Foot ulcers repair
Restore	DePuy Orthopaedics	Soft tissue reinforcement
Surgisis	Cook Surgical	Hernias repair
Durepair	TEI Bio-sciences	Dura mater repair
Osteomesh	Osteopore International	Craniofacial repair
Bio-Oss	Geistlich Pharma AG	Alveolar bone reconstruction
BoneCeramic	Institut Straumann AG	Alveolar bone reconstruction
Natix	Tigran Tech AB	Alveolar bone reconstruction
Puros	Zimmer Biomet	Periodontal and alveolar bone reconstructive
Tactoset	Anika	Bone marrow lesions or insufficiency fractures via percutaneous skeletal fixation
Hyalofast	Anika	Repair of chondral and osteochondral lesions

#### Random structure mimetic biomaterials

The ECMs of most human organs exhibits random structures. For example, the collagen fibers present in the dermis and subcutaneous connective tissue show a random structure [212]. Thus, randomly oriented nanofiber substrates produced by electrospinning and decellularization can be used for wound healing. Sun et al. [293] reported that random electrospun poly(ɛ-caprolactone) (PCL) nanofiber membranes could be used as a wound dressing that enhanced diabetic wound healing. However, these nanofiber membranes are only suitable for superficial wounds, while most skin injuries include dermal and subcutaneous connective tissue defects or volumetric tissue loss. Chen et al. [294] reported a 3D short nanofiber scaffold fabricated by combining freeze-drying and electrospinning. The resulting structure was similar to endogenous ECM collagen. Fibroblasts cultured on such scaffolds exhibited cell infiltration and proliferation in vitro. Compared to synthetic and semisynthetic skin equivalents, the acellular dermal matrix (ADM) is still considered the most promising alternative substrate for replacing the damaged dermis [295].

#### Aligned structure mimetic biomaterials

Although randomly structured ECMs are common, several anatomical sites have aligned ECMs (e.g., tendon [282], muscle [117]). To more accurately mimic aligned ECMs, Choi et al. [296] prepared an aligned PCL/collagen-based nanofiber scaffold by electrospinning. These scaffolds were used for culturing human skeletal muscle cells to repair volumetric muscle loss. The aligned nanofibers significantly improved the orientation of muscle cells and myotube formation relative to random nanofibers [296]. In addition to mimicking the naturally aligned ECM, aligned scaffolds can be used to enhance cell migration into a defect sight, leading to faster tissue regeneration. The infiltration of cells (e.g., fibroblast, endothelial cells, keratinocytes) is usually limited in scaffolds without directionality. However, since uniaxial-aligned nanofibrous scaffolds can guide and align cells from both ends, enhanced granular tissue formation, angiogenesis and re-epithelialization can be achieved in wound models [297]. Radially aligned nanofiber scaffolds may have more advantages in promoting cell migration. Xie et al. [298] fabricated a radially aligned PCL nanofiber membrane by utilizing a collector composed of a peripheral ring electrode and a central point electrode. This structure accelerated cell migration from their periphery to the center of the scaffolds. Similarly, Chen et al. [299] developed a 3D radially aligned PCL nanofiber scaffold by combining electrospinning, thermo-fixation, and gas foaming. The 3D radial nanofiber scaffolds had very high porosity, consisting of layers of aligned nanofibers separated by gaps ranging from several micrometers to several millimeters.

#### Random-to-aligned gradient structure mimetic biomaterials

Tendons are the connective tissues that bridge muscle to bone and allow transmission of forces to produce joint movement. Collagen fiber bundles present at the transition from the bone to tendon show a unique random-to-aligned structure. Thus, Xie et al. [300] prepared “random-to-aligned” electrospun PCL nanofiber membranes to mimic the orientation of collagen fibers at the tendon-to-bone insertion site. Tendon fibroblasts seeded on such membranes exhibited highly organized and haphazardly oriented morphologies on the aligned and random areas, respectively, thereby mimicking the desired anatomy in both form and function.

#### Porous structure mimetic biomaterials

Pore size and scaffold porosity influence the focal adhesion, migration, and proliferation of cells. Although bioceramics are promising bone replacement materials, their small and unconnected pores limit their applications [217, 285]. To address this, Dapporto et al. [301] introduced air bubbles of tailored volume and size in the ceramic suspensions, resulting in HAP scaffolds with open and interconnected pores. Porous biomaterials are a prerequisite for hard and soft tissue repair [18, 38, 55, 121, 218]. Recently, aerogels consisting of short nanofibers have been explored for a variety of TE applications. Although the dense structure of aerogels generally prevents cell penetration, John et al. [302] created anisotropic micro-channels and patterned macro-channels within the short nanofiber aerogels by freeze-casting of 3D-printed templates. The in vitro experiments demonstrated that the final macro-/micro-channel structure could significantly increase preosteoblast infiltration. After subcutaneous implantation in vivo, these aerogels promoted faster cellular penetration and better tissue integration with host tissue compared to aerogels without the macro-channel structure.

#### Gradient porous structure mimetic biomaterials

Several anatomies exhibit gradient porous structures [1]. For instance, bones increase in porosity from the periosteum inward [26]. Accordingly, designing scaffolds with gradient porous structures may be an appealing approach to regenerating such tissues. Several methods have been utilized to design such scaffolds, whereby centrifugation is arguably the easiest approach for creating a gradient porous structure. Oh et al. [303]. utilized centrifugation to fabricate a PCL cylindrical scaffold with gradually increasing pore size along the longitudinal axis. The PCL scaffold with gradient pore size distribution served as an ideal substrate to explore the gradual change of pore size on cell differentiation [303]. Zhang et al. [304] developed a collagen scaffold with a gradient pore size distribution from 150 to 500 μm by utilizing ice particles as a porogen. Moreover, all pores were connected, allowing for cell migration and distribution, so that the collagen scaffolds were able to promote cartilage regeneration. Xie et al. [298] developed another 3D nanofiber scaffold with a gradient pore size distribution by using a combination of electrospinning, modified gas foaming, and gradient surfactant incorporation. The pore sizes gradually increased from 100 to 1700 μm and were correlated with the gradient increase of surfactant concentration. The scaffolds with a gradient pore size distribution are able to create microenvironments with different oxygen concentrations. Areas in the scaffold with minimal surfactant had dense structures with small pore size and easily formed a hypoxic environment. While hypoxic conditions are good for osteogenic differentiation, high structures with a large pore size are optimal for chondrogenic differentiation [223].

### Applications of function-mimetic biomaterials

#### Conductive biomaterials

Electrochemical signaling plays a significant role in the functioning of the human body and is present in nerves [252], skeletal muscles [117], and hearts [124, 266]. Conductive biomaterials were developed to enhance electrochemical cell functions by amplifying cellular signaling, particularly when tissues are damaged. For instance, Xu et al. [305] synthesized a conductive nerve conduit based on polypyrrole (PPY)/PDLLA. These PPY/PDLLA scaffolds were able to increase neurite length and percentage of neurite-bearing cells of rat adrenal pheochromocytoma PC12 cells under electro-simulation. Moreover, the resulting PPY/PDLLA nerve conduits could repair a rat sciatic nerve defect in rats, and the functional outcomes were similar to autologous grafts, which are considered the current gold standard. In addition to nerve regeneration, conductive biomaterials are also good candidates for muscle regeneration, since muscle tissues respond to electrochemical signals. For example, Dong et al. [117] developed an elastic conductive poly (ethylene glycol)-co-poly (glycerol sebacate) grafted aniline pentamer copolymer, which could promote the proliferation of murine skeletal muscle C2C12 cells and enhance the formation of myotubes.

#### Viscoelastic biomaterials

The ECM of living tissue exhibit viscoelasticity, which affects cell and tissue behaviors [306]. As such, efforts toward developing viscoelastic TE scaffolds, particularly for minimally invasive surgery, have increased. For example, Costa et al. [307] fabricated a highly elastic hybrid scaffold for meniscus regeneration by co-printing a cell-laden fibrinogen/gellan gum composite bioink with a methacrylate-modified silk fibroin bioink. The viscoelastic scaffolds provided an ideal artificial substrate and exhibited excellent biomechanical properties for maintaining porcine primary meniscus cells. In addition, the fibrocartilaginous tissue formed without changing its shape during the 10-week post implantation period because of its viscoelastic properties. Since cardiac tissue constantly undulates between relaxed and stretched states, artificial heart scaffolds must have super-elastic properties. To this end, Davenport Huyer et al. [308] synthesized a poly [octamethylene maleate (anhydride) 1,2,4-butanetricarboxylate] pre-polymer gel in a one-step polycondensation reaction. The resulting scaffolds improved the elastic properties of polyester biomaterials for cardiac TE applications. Its elastomeric properties fell within the range of adult myocardium, and it could support rat cardiac cell attachment in vitro.

#### Thermosensitive biomaterials

Among the different types of smart biomaterials, which can respond to different signals such as temperature, light, pH, magnetism, etc., thermosensitive hydrogels are perhaps one of the most widely explored, as they have great potential for applications in tissue regeneration and drug release. Generally, thermosensitive hydrogels contain both hydrophilic and hydrophobic components. Temperature changes can switch the interaction between hydrophobic and hydrophilic segments, resulting in changes of the solubility of the cross-linked network, which can trigger the sol-gel transition [309].

Chung et al. [310] synthesized two kinds of thermosensitive chitosan copolymers, including the N-isopropylacrylamide (NIPAAm)-grafted chitosan and Pluronic®-modified chitosan. These hybrid materials could form thermally reversible hydrogels that supported human mesenchymal stem cell attachment and chondrogenic differentiation. These thermoresponsive scaffolds have great application potential as injectable cartilage repair material. As another major kind of thermosensitive material, PEG has excellent biocompatibility and has been widely used in the design and fabrication of thermoresponsive hydrogels. A series of biodegradable and thermosensitive PEG hydrogels with adjustable properties have been developed for tissue regeneration and drug release by incorporating hydrophobic segments, such as polypeptides and polyesters [311].

#### pH sensitive biomaterials

Most human organs have a neutral pH, but some tissues have significantly altered pH values. For example, the stomach has a strong acidity while the intestinal has a basic pH value. Thus, biomaterials for stomach and intestine wound healing or disease treatment should match the local pH microenvironment. He et al. [312] developed a variety of injectable pH-responsive adhesive hydrogels based on the AA-g-N-hydroxysuccinimide and acryloyl-6-aminocaproic acid. This hydrogel showed good biocompatibility, fast gelation time, self-healing ability, and hemostatic properties under a strongly acidic condition. Furthermore, enhanced swine gastric wound healing was observed along with blood vessel formation, collagen accumulation and α-smooth muscle actin (α-SMA) expression.

## Conclusions and prospects

Integrated biomimetic TE scaffolds containing natural biomaterials are gaining increasing attention due to their inherent advantages over conventional TE scaffolds materials. Notably, natural biomaterials have inherently biocompatible compositions, microstructures and bioactivity similarity to the native ECM. In addition, natural biomaterials may have tunable shapes, mechanical properties, designer characteristics, and versatility. These characteristics, combined with corresponding biomimetic design and adaptation of exterior (bio-)chemistry, structure, and properties, enable the adaptation of natural biomaterials as smart and versatile platforms for the design and engineering of scaffolds that allow controlled drug delivery and efficient tissue regeneration. Biomimetic scaffolds based on natural biomaterials offer a promising basis for the repair and regeneration of a wide range of tissues, including myocardium, skeletal muscle, peripheral nerves, cartilage, ligaments, cornea, skin and bone.

However, despite significant advances in BNBMs, their clinical applications have proven to be very challenging, and the development of improved therapeutics based on BNBMs is still an area of active research. To effectively utilize the BNBMs for clinical prosthetics, in-depth research needs to be implemented to optimize the scaffold-cell interactions, controllable degradation, stress response, and adaptability of the scaffold. Other challenges include finding a suitable balance between the resistance to degradation and to decreased immunogenicity, while maintaining natural mechanical characteristics and other physicochemical properties. Despite numerous studies demonstrating optimal performance of such scaffolds in vitro or in animal models, it is unclear how well many of these materials will translate to use in human patients. There is therefore still a long way to go before we can realize the translation from experimental TE scaffolds to clinically applied technology. Anyway, the biomimetic functionality of natural-origin biomaterials not only represents novel opportunities to fix complex physicochemical, physiological, and biological pathologies in distinct tissues, but may also help us gain a deeper understanding of diverse biological activities and properties of the ECM and its components.

## Data Availability

The data and materials used during the current review are all available in this review.

## Abbreviations

3D: Three-dimensional
3HB: 3-Hydroxybutyrate
*A.** vinelandii*: *Azotobacter vinelandii*
*adhE*: *alcohol dehydrogenase*
ADM: Acellular dermal matrix
AI: Artificial intelligence
AMP: Antimicrobial peptide
*B*. *subtilis*: *Bacillus subtilis*
BC: Bacterial cellulose
BNBMs: Biomimetic natural biomaterials
*C*. *glutamate*: Corynebacterium glutamicum
*C*. *glutamicum*: Corynebacterium glutamicum
*C*. *reinhardtii*: Chlamydomonas reinhardtii
CMCS: Carboxymethyl chitosan
CRISPR-Cas9: Clustered regularly interspaced short palindromic repeats-CRISPR associated protein 9
dECM: Decellularized ECM
DTT: Dithiothreitol
*E. coli*: *Escherichia coli*
ECM: Extracellular matrix
*FadA*: *Acetyl-CoA C-acyltransferase*
*FadB*: *Fatty acid oxidation complex subunit alpha*
*FtsZ*: *Cell division protein*
*GabD*: *Succinate-semialdehyde dehydrogenase*
GelMA: Gelatin Methacrylatemethacrylate gelatin
GSH: Glutathione
*H.** bluephagenesis*: *Halomonas bluephagenesis*
HA: Hyaluronic acid
HAP: Hydroxyapatite
hBD-2: Human beta-defensin 2
hLYZ: Human lysozyme
IPN: Interpenetrating polymer network
LA: Lactic acid
MCC: 2-methylcitrate cycle
MEC: Mechano-electrical coupling
ML: Machine learning
MNP: Magnetic nanoparticle
MOF: Metal-organic framework
*MreB*: *Rod shape-determining protein*
NBMs: Natural biomaterials
NIPAAm: N-isopropylacrylamide
PAA: Polyacrylic acid
PCL: Poly(ɛ-caprolactone)
PDLLA: Poly (D, L-lactide)
PEG: Polyethylene glycol
PEO: Polyethylene oxide
PGA: Polyglutamic acids
PHA: Polyhydroxyalkanoate
PHB: Polyhydroxybutyrate
PHBV: P(3-hydroxybutyrate-co-3-hydroxyvalerate)
PLA: Polylactic acid
PLGA: Poly (lactic-co-glycolic acid)
PPY: Polypyrrole
PVA: Polyvinyl alcohol
PVDF: Polyvinylidene difluoride
rCols: Recombinant collagens
*S.** aureus*: *Staphylococcus aureus*
*S. cerevisiae*: *Saccharomyces cerevisiae*
SA: Sodium alginate
TE: Tissue engineering
Tg: Glass transition temperature
*UdhA*: *Pyridine nucleotide transhydrogenase*
UV: Ultraviolet
VEGF: Vascular endothelial growth factor
α-SMA: α-smooth muscle actin

## References

[1] Naik RR, Singamaneni S (2017). Introduction: bioinspired and biomimetic materials. Chem Rev.

[2] Huang G, Li F, Zhao X, Ma Y, Li Y, Lin M (2017). Functional and biomimetic materials for engineering of the three-dimensional cell microenvironment. Chem Rev.

[3] Shin H, Jo S, Mikos AG (2003). Biomimetic materials for tissue engineering. Biomaterials.

[4] Ullah S, Chen X (2020). Fabrication, applications and challenges of natural biomaterials in tissue engineering. Appl Mater Today.

[5] Sheikh Z, Hamdan N, Ikeda Y, Grynpas M, Ganss B, Glogauer M (2017). Natural graft tissues and synthetic biomaterials for periodontal and alveolar bone reconstructive applications: a review. Biomater Res.

[6] Insuasti-Cruz E, Suárez-Jaramillo V, Mena Urresta KA, Pila-Varela KO, Fiallos-Ayala X, Dahoumane SA (2022). Natural biomaterials from biodiversity for healthcare applications. Adv Healthc Mater.

[7] Garlotta D (2001). A literature review of poly (lactic acid). J Polym Environ.

[8] Lasprilla AJR, Martinez GAR, Lunelli BH, Jardini AL, Filho RM (2012). Poly-lactic acid synthesis for application in biomedical devices—a review. Biotechnol Adv.

[9] Andhariya JV, Burgess DJ (2016). Recent advances in testing of microsphere drug delivery systems. Expert Opin Drug Deliv.

[10] Hua Y, Su Y, Zhang H, Liu N, Wang Z, Gao X (2021). Poly(lactic-co-glycolic acid) microsphere production based on quality by design: a review. Drug Deliv.

[11] Lamprecht A, Ubrich N, Hombreiro Pérez M, Lehr C, Hoffman M, Maincent P (2000). Influences of process parameters on nanoparticle preparation performed by a double emulsion pressure homogenization technique. Int J Pharm.

[12] Bolland BJRF, Kanczler JM, Ginty PJ, Howdle SM, Shakesheff KM, Dunlop DG (2008). The application of human bone marrow stromal cells and poly(dl-lactic acid) as a biological bone graft extender in impaction bone grafting. Biomaterials.

[13] Milan J-L, Planell JA, Lacroix D (2009). Computational modelling of the mechanical environment of osteogenesis within a polylactic acid-calcium phosphate glass scaffold. Biomaterials.

[14] Pavot V, Berthet M, Rességuier J, Legaz S, Handké N, Gilbert SC (2014). Poly(lactic acid) and poly(lactic-co-glycolic acid) particles as versatile carrier platforms for vaccine delivery. Nanomedicine (Lond).

[15] Lee W, Park J (2012). The design of a heterocellular 3D architecture and its application to monitoring the behavior of cancer cells in response to the spatial distribution of endothelial cells. Adv Mater.

[16] Chen GQ, Jiang XR (2018). Engineering microorganisms for improving polyhydroxyalkanoate biosynthesis. Curr Opin Biotechnol.

[17] Chen GQ (2009). A microbial polyhydroxyalkanoates (PHA) based bio- and materials industry. Chem Soc Rev.

[18] Wei DX, Dao JW, Chen GQ (2018). A micro-ark for cells: Highly open porous polyhydroxyalkanoate microspheres as injectable scaffolds for tissue regeneration. Adv Mater.

[19] Wei DX, Dao JW, Liu HW, Chen GQ (2018). Suspended polyhydroxyalkanoate microspheres as 3D carriers for mammalian cell growth. Artif Cells Nanomed Biotechnol.

[20] Zhao XH, Peng XL, Gong HL, Wei DX (2021). Osteogenic differentiation system based on biopolymer nanoparticles for stem cells in simulated microgravity. Biomed Mater.

[21] Chen R, Yu J, Gong HL, Jiang Y, Xue M, Xu N (2020). An easy long-acting BMP7 release system based on biopolymer nanoparticles for inducing osteogenic differentiation of adipose mesenchymal stem cells. J Tissue Eng Regen Med.

[22] Hu J, Wang M, Xiao X, Zhang B, Xie Q, Xu X (2020). A novel long-acting azathioprine polyhydroxyalkanoate nanoparticle enhances treatment efficacy for systemic lupus erythematosus with reduced side effects. Nanoscale.

[23] Peng X-L, Cheng J-S-Y, Gong H-L, Yuan M-D, Zhao X-H, Li Z (2021). Advances in the design and development of SARS-CoV-2 vaccines. Mil Med Res.

[24] Wang ZH, Zhang J, Zhang Q, Gao Y, Yan J, Zhao XY (2016). Evaluation of bone matrix gelatin/fibrin glue and chitosan/gelatin composite scaffolds for cartilage tissue engineering. Genet Mol Res.

[25] Wang Z-Y, Zhang X-W, Ding Y-W, Ren Z-W, Wei D-X (2023). Natural biopolyester microspheres with diverse structures and surface topologies as micro-devices for biomedical applications. Smart Mater Med.

[26] Ding Y-W, Zhang X-W, Mi C-H, Qi X-Y, Zhou J, Wei D-X (2023). Recent advances in hyaluronic acid-based hydrogels for 3D bioprinting in tissue engineering applications. Smart Mater Med.

[27] Sunguroğlu C, Sezgin DE, Aytar Çelik P, Çabuk A (2018). Higher titer hyaluronic acid production in recombinant Lactococcus lactis. Prep Biochem Biotechnol.

[28] Jeong E, Shim WY, Kim JH (2014). Metabolic engineering of Pichia pastoris for production of hyaluronic acid with high molecular weight. J Biotechnol.

[29] Sze JH, Brownlie JC, Love CA (2016). Biotechnological production of hyaluronic acid: a mini review. 3 Biotech.

[30] Ding Y-W, Wang Z-Y, Ren Z-W, Zhang X-W, Wei D-X (2022). Advances in modified hyaluronic acid-based hydrogels for skin wound healing. Biomater Sci.

[31] Wang L, Sun L, Bian F, Wang Y, Zhao Y (2022). Self-bonded hydrogel inverse opal particles as sprayed flexible patch for wound healing. ACS Nano.

[32] Ma W, Zhang X, Liu Y, Fan L, Gan J, Liu W (2022). Polydopamine decorated microneedles with Fe-MSC-derived nanovesicles encapsulation for wound healing. Adv Sci (Weinh).

[33] Zhou J, Zhang B, Liu X, Shi L, Zhu J, Wei D (2016). Facile method to prepare silk fibroin/hyaluronic acid films for vascular endothelial growth factor release. Carbohydr Polym.

[34] George M, Abraham TE (2006). Polyionic hydrocolloids for the intestinal delivery of protein drugs: alginate and chitosan–a review. J Control Release.

[35] Wee S, Gombotz WR (1998). Protein release from alginate matrices. Adv Drug Deliv Rev.

[36] Fernando IPS, Kim D, Nah J-W, Jeon Y-J (2019). Advances in functionalizing fucoidans and alginates (bio)polymers by structural modifications: a review. Chem Eng J.

[37] Fan L, Hu L, Xie J, He Z, Zheng Y, Wei D (2021). Biosafe, self-adhesive, recyclable, tough, and conductive hydrogels for multifunctional sensors. Biomater Sci.

[38] Fu S, Du X, Zhu M, Tian Z, Wei D, Zhu Y (2019). 3D printing of layered mesoporous bioactive glass/sodium alginate-sodium alginate scaffolds with controllable dual-drug release behaviors. Biomed Mater.

[39] Shapiro L, Cohen S (1997). Novel alginate sponges for cell culture and transplantation. Biomaterials.

[40] Kang E, Choi YY, Chae SK, Moon JH, Chang JY, Lee SH (2012). Microfluidic spinning of flat alginate fibers with grooves for cell-aligning scaffolds. Adv Mater.

[41] Song X, Wang X, Zhang J, Shen S, Yin W, Ye G (2021). A tunable self-healing ionic hydrogel with microscopic homogeneous conductivity as a cardiac patch for myocardial infarction repair. Biomaterials.

[42] Borges AL, Castro B, Govindarajan S, Solvik T, Escalante V, Bondy-Denomy J (2020). Bacterial alginate regulators and phage homologs repress CRISPR-Cas immunity. Nat Microbiol.

[43] Van Rensburg P, Van Zyl WH, Pretorius IS (1998). Engineering yeast for efficient cellulose degradation. Yeast.

[44] Zhou S, Nyholm L, Strømme M, Wang Z (2019). Cladophora cellulose: Unique biopolymer nanofibrils for emerging energy, environmental, and life science applications. Acc Chem Res.

[45] Klemm D, Heublein B, Fink HP, Bohn A (2005). Cellulose: fascinating biopolymer and sustainable raw material. Angew Chem Int Ed Engl.

[46] Chen GQ, Jiang XR (2018). Next generation industrial biotechnology based on extremophilic bacteria. Curr Opin Biotechnol.

[47] Bodin A, Bharadwaj S, Wu S, Gatenholm P, Atala A, Zhang Y (2010). Tissue-engineered conduit using urine-derived stem cells seeded bacterial cellulose polymer in urinary reconstruction and diversion. Biomaterials.

[48] He Y, Hou H, Wang S, Lin R, Wang L, Yu L (2021). From waste of marine culture to natural patch in cardiac tissue engineering. Bioact Mater.

[49] Yi H, Wu LQ, Bentley WE, Ghodssi R, Rubloff GW, Culver JN (2005). Biofabrication with chitosan. Biomacromol.

[50] Bellich B, D'Agostino I, Semeraro S, Gamini A, Cesàro A (2016). The good, the bad and the ugly of chitosans. Mar Drugs.

[51] Santos JCC, Moreno PMD, Mansur AAP, Leiro V, Mansur HS, Pêgo AP (2015). Functionalized chitosan derivatives as nonviral vectors: physicochemical properties of acylated N, N, N-trimethyl chitosan/oligonucleotide nanopolyplexes. Soft Matter.

[52] Huang M, Khor E, Lim L-Y (2004). Uptake and cytotoxicity of chitosan molecules and nanoparticles: effects of molecular weight and degree of deacetylation. Pharm Res.

[53] Saranya N, Moorthi A, Saravanan S, Devi MP, Selvamurugan N (2011). Chitosan and its derivatives for gene delivery. Int J Biol Macromol.

[54] Xu T, Yang H, Yang D, Yu Z-Z (2017). Polylactic acid nanofiber scaffold decorated with chitosan islandlike topography for bone tissue engineering. ACS Appl Mater Interfaces.

[55] Ullah S, Zainol I, Chowdhury SR, Fauzi MB (2018). Development of various composition multicomponent chitosan/fish collagen/glycerin 3D porous scaffolds: effect on morphology, mechanical strength, biostability and cytocompatibility. Int J Biol Macromol.

[56] Li P, Liu S, Yang X, Du S, Tang W, Cao W (2021). Low-drug resistance carbon quantum dots decorated injectable self-healing hydrogel with potent antibiofilm property and cutaneous wound healing. Chem Eng J.

[57] Buehler MJ (2006). Nature designs tough collagen: explaining the nanostructure of collagen fibrils. Proc Natl Acad Sci U S A.

[58] Sorushanova A, Delgado LM, Wu Z, Shologu N, Kshirsagar A, Raghunath R (2019). The collagen suprafamily: from biosynthesis to advanced biomaterial development. Adv Mater.

[59] Wu H, Zhang R, Hu B, He Y, Zhang Y, Cai L (2021). A porous hydrogel scaffold mimicking the extracellular matrix with swim bladder derived collagen for renal tissue regeneration. Chin Chem Lett.

[60] Huang W, Ling S, Li C, Omenetto FG, Kaplan DL (2018). Silkworm silk-based materials and devices generated using bio-nanotechnology. Chem Soc Rev.

[61] Gatesy J, Hayashi C, Motriuk D, Woods J, Lewis R (2001). Extreme diversity, conservation, and convergence of spider silk fibroin sequences. Science.

[62] Wei S, Ma J-X, Xu L, Gu X-S, Ma X-L (2020). Biodegradable materials for bone defect repair. Mil Med Res.

[63] Liu S, Pu Y, Yang R, Liu X, Wang P, Wang X (2020). Boron-assisted dual-crosslinked poly (γ-glutamic acid) hydrogels with high toughness for cartilage regeneration. Int J Biol Macromol.

[64] Liu X, Liu S, Yang R, Wang P, Zhang W, Tan X (2021). Gradient chondroitin sulfate/poly (γ-glutamic acid) hydrogels inducing differentiation of stem cells for cartilage tissue engineering. Carbohydr Polym.

[65] Sirisansaneeyakul S, Cao M, Kongklom N, Chuensangjun C, Shi Z, Chisti Y (2017). Microbial production of poly-γ-glutamic acid. World J Microbiol Biotechnol.

[66] Xu G, Zha J, Cheng H, Ibrahim MHA, Yang F, Dalton H (2019). Engineering Corynebacterium glutamicum for the de novo biosynthesis of tailored poly-γ-glutamic acid. Metab Eng.

[67] Lee J-K, Luchian T, Park Y (2018). New antimicrobial peptide kills drug-resistant pathogens without detectable resistance. Oncotarget.

[68] Montalvo GEB, Vandenberghe LPDS, Soccol VT, Carvalho JCD, Soccol CR (2020). The antihypertensive, antimicrobial and anticancer peptides from Arthrospira with therapeutic potential: a mini review. Curr Mol Med.

[69] Zhang Q-Y, Yan Z-B, Meng Y-M, Hong X-Y, Shao G, Ma J-J (2021). Antimicrobial peptides: mechanism of action, activity and clinical potential. Mil Med Res.

[70] Gong T, Fu J, Shi L, Chen X, Zong X (2021). Antimicrobial peptides in gut health: a review. Front Nutr.

[71] Wei D, Zhang X (2022). Biosynthesis, bioactivity, biotoxicity and applications of antimicrobial peptides for human health. Biosaf Health.

[72] Tan D, Xue Y-S, Aibaidula G, Chen G-Q (2011). Unsterile and continuous production of polyhydroxybutyrate by Halomonas TD01. Bioresour Technol.

[73] Song Y, Matsumoto KI, Yamada M, Gohda A, Brigham CJ, Sinskey AJ (2012). Engineered Corynebacterium glutamicum as an endotoxin-free platform strain for lactate-based polyester production. Appl Microbiol Biotechnol.

[74] Cheng F, Luozhong S, Guo Z, Yu H, Stephanopoulos G (2017). Enhanced biosynthesis of hyaluronic acid using engineered Corynebacterium glutamicum via metabolic pathway regulation. Biotechnol J.

[75] Matsumoto KI, Tobitani K, Aoki S, Song Y, Ooi T, Taguchi S (2014). Improved production of poly(lactic acid)-like polyester based on metabolite analysis to address the rate-limiting step. AMB Express.

[76] Singh A, Walker KT, Ledesma-Amaro R, Ellis T (2020). Engineering bacterial cellulose by synthetic biology. Int J Mol Sci.

[77] Desai SK, Gallivan JP (2004). Genetic screens and selections for small molecules based on a synthetic riboswitch that activates protein translation. J Am Chem Soc.

[78] Suess B, Fink B, Berens C, Stentz R, Hillen W (2004). A theophylline responsive riboswitch based on helix slipping controls gene expression in vivo. Nucleic Acids Res.

[79] Bayer TS, Smolke CD (2005). Programmable ligand-controlled riboregulators of eukaryotic gene expression. Nat Biotechnol.

[80] Werstuck G, Green MR (1998). Controlling gene expression in living cells through small molecule-RNA interactions. Science.

[81] Tao W, Lv L, Chen GQ (2017). Engineering Halomonas species TD01 for enhanced polyhydroxyalkanoates synthesis via CRISPRi. Microb Cell Fact.

[82] Lv L, Ren YL, Chen JC, Wu Q, Chen GQ (2015). Application of CRISPRi for prokaryotic metabolic engineering involving multiple genes, a case study: Controllable P(3HB-co-4HB) biosynthesis. Metab Eng.

[83] Widner B, Behr R, Von Dollen S, Tang M, Heu T, Sloma A (2005). Hyaluronic acid production in Bacillus subtilis. Appl Environ Microbiol.

[84] Zhang X, Xia L, Day BA, Harris TI, Oliveira P, Knittel C (2019). CRISPR/Cas9 initiated transgenic silkworms as a natural spinner of spider silk. Biomacromol.

[85] Liu X, Wang Y, Tian Y, Yu Y, Gao M, Hu G (2014). Generation of mastitis resistance in cows by targeting human lysozyme gene to β-casein locus using zinc-finger nucleases. Proc Biol Sci.

[86] Martemyanov KA, Shirokov VA, Kurnasov OV, Gudkov AT, Spirin AS (2001). Cell-free production of biologically active polypeptides: application to the synthesis of antibacterial peptide cecropin. Protein Expr Purif.

[87] Li T, Ye J, Shen R, Zong Y, Zhao X, Lou C (2016). Semirational approach for ultrahigh poly(3-hydroxybutyrate) accumulation in Escherichia coli by combining one-step library construction and high-throughput screening. ACS Synth Biol.

[88] Cheng F, Gong Q, Yu H, Stephanopoulos G (2016). High-titer biosynthesis of hyaluronic acid by recombinant Corynebacterium glutamicum. Biotechnol J.

[89] Zhao H, Zhang HM, Chen X, Li T, Wu Q, Ouyang Q (2017). Novel T7-like expression systems used for Halomonas. Metab Eng.

[90] Liu W, Lin H, Zhao P, Xing L, Li J, Wang Z (2022). A regulatory perspective on recombinant collagen-based medical devices. Bioact Mater.

[91] Wei XX, Shi ZY, Yuan MQ, Chen GQ (2009). Effect of anaerobic promoters on the microaerobic production of polyhydroxybutyrate (PHB) in recombinant Escherichia coli. Appl Microbiol Biotechnol.

[92] Li ZJ, Shi ZY, Jian J, Guo YY, Wu Q, Chen GQ (2010). Production of poly(3-hydroxybutyrate-co-4-hydroxybutyrate) from unrelated carbon sources by metabolically engineered Escherichia coli. Metab Eng.

[93] Fu X-Z, Tan D, Aibaidula G, Wu Q, Chen JC, Chen GQ (2014). Development of Halomonas TD01 as a host for open production of chemicals. Metab Eng.

[94] Tan D, Wu Q, Chen JC, Chen GQ (2014). Engineering Halomonas TD01 for the low-cost production of polyhydroxyalkanoates. Metab Eng.

[95] Yin J, Chen JC, Wu Q, Chen GQ (2015). Halophiles, coming stars for industrial biotechnology. Biotechnol Adv.

[96] Wang Y, Wu H, Jiang X, Chen GQ (2014). Engineering *Escherichia col*i for enhanced production of poly(3-hydroxybutyrate-co-4-hydroxybutyrate) in larger cellular space. Metab Eng.

[97] Wang Z, Qin Q, Zheng Y, Li F, Zhao Y, Chen GQ (2021). Engineering the permeability of Halomonas bluephagenesis enhanced its chassis properties. Metab Eng.

[98] Castillo T, Heinzle E, Peifer S, Schneider K, Peña MCF (2013). Oxygen supply strongly influences metabolic fluxes, the production of poly(3-hydroxybutyrate) and alginate, and the degree of acetylation of alginate in Azotobacter vinelandii. Process Biochem.

[99] Guo J, Luo YE, Fan D, Yang B, Gao P, Ma X (2010). Medium optimization based on the metabolic-flux spectrum of recombinant Escherichia coli for high expression of human-like collagen II. Biotechnol Appl Biochem.

[100] Zhong C, Zhang GC, Liu M, Zheng XT, Han PP, Jia S-R (2013). Metabolic flux analysis of Gluconacetobacter xylinus for bacterial cellulose production. Appl Microbiol Biotechnol.

[101] Zheng Y, Cheng F, Zheng B, Yu H (2020). Enhancing single-cell hyaluronic acid biosynthesis by microbial morphology engineering. Synth Syst Biotechnol.

[102] Lata S, Sharma BK, Raghava GPS (2007). Analysis and prediction of antibacterial peptides. BMC Bioinform.

[103] Fjell CD, Jenssen H, Hilpert K, Cheung WA, Panté N, Hancock REW (2009). Identification of novel antibacterial peptides by chemoinformatics and machine learning. J Med Chem.

[104] Zhou Y, Li G, Dong J, Xing XH, Dai J, Zhang C (2018). MiYA, an efficient machine-learning workflow in conjunction with the YeastFab assembly strategy for combinatorial optimization of heterologous metabolic pathways in Saccharomyces cerevisiae. Metab Eng.

[105] Jervis AJ, Carbonell P, Taylor S, Sung R, Dunstan MS, Robinson CJ (2019). SelProm: a queryable and predictive expression vector selection tool for *Escherichia*
*coli*. ACS Synth Biol.

[106] Yang KK, Wu Z, Arnold FH (2019). Machine-learning-guided directed evolution for protein engineering. Nat Methods.

[107] Hadadi N, Hatzimanikatis V (2015). Design of computational retrobiosynthesis tools for the design of de novo synthetic pathways. Curr Opin Chem Biol.

[108] Manfrão-Netto JHC, Queiroz EB, de Oliveira Junqueira AC, Gomes AMV, Gusmão De Morais D, Paes HC (2022). Genetic strategies for improving hyaluronic acid production in recombinant bacterial culture. J Appl Microbiol.

[109] Bejagam KK, Lalonde J, Iverson CN, Marrone BL, Pilania G (2022). Machine learning for melting temperature predictions and design in polyhydroxyalkanoate-based biopolymers. J Phys Chem B.

[110] Pilania G, Iverson CN, Lookman T, Marrone BL (2019). Machine-learning-based predictive modeling of glass transition temperatures: a case of polyhydroxyalkanoate homopolymers and copolymers. J Chem Inf Model.

[111] Xu RZ, Cao JS, Luo JY, Feng Q, Ni BJ, Fang F (2022). Integrating mechanistic and deep learning models for accurately predicting the enrichment of polyhydroxyalkanoates accumulating bacteria in mixed microbial cultures. Bioresour Technol.

[112] Pazhamannil RV, Govindan P, Sooraj P (2021). Prediction of the tensile strength of polylactic acid fused deposition models using artificial neural network technique. Mater Today.

[113] Zhang Y, Xu JL, Yuan ZH. Modeling and prediction in the enzymatic hydrolysis of cellulose using artificial neural networks. In: 2009 fifth international conference on natural computation: IEEE, 2009; pp. 158–62

[114] Rodríguez-Dorado R, Landín M, Altai A, Russo P, Aquino RP, Del Gaudio P (2018). A novel method for the production of core-shell microparticles by inverse gelation optimized with artificial intelligent tools. Int J Pharm.

[115] Damiati SA, Rossi D, Joensson HN, Damiati S (2020). Artificial intelligence application for rapid fabrication of size-tunable PLGA microparticles in microfluidics. Sci Rep.

[116] da Silva E, Silva N, de Souza FF, dos Santos Freitas MM, Pino Hernández EJG, Dantas VV, Enê Chaves Oliveira M (2021). Artificial intelligence application for classification and selection of fish gelatin packaging film produced with incorporation of palm oil and plant essential oils. Food Packag Shelf Life.

[117] Dong R, Zhao X, Guo B, Ma PX (2017). Biocompatible elastic conductive films significantly enhanced myogenic differentiation of myoblast for skeletal muscle regeneration. Biomacromolecules.

[118] Fabbro A, Scaini D, León V, Vázquez E, Cellot G, Privitera G (2016). Graphene-based interfaces do not alter target nerve cells. ACS Nano.

[119] Ryan AJ, Kearney CJ, Shen N, Khan U, Kelly AG, Probst C (2018). Electroconductive biohybrid collagen/pristine graphene composite biomaterials with enhanced biological activity. Adv Mater.

[120] He Y, Ye G, Song C, Li C, Xiong W, Yu L (2018). Mussel-inspired conductive nanofibrous membranes repair myocardial infarction by enhancing cardiac function and revascularization. Theranostics.

[121] Song X, Mei J, Ye G, Wang L, Ananth A, Yu L (2019). In situ pPy-modification of chitosan porous membrane from mussel shell as a cardiac patch to repair myocardial infarction. Appl Mater Today.

[122] Dvir T, Timko BP, Brigham MD, Naik SR, Karajanagi SS, Levy O (2011). Nanowired three-dimensional cardiac patches. Nat Nanotechnol.

[123] Wang L, Liu Y, Ye G, He Y, Li B, Guan Y (2021). Injectable and conductive cardiac patches repair infarcted myocardium in rats and minipigs. Nat Biomed Eng.

[124] Xiong W, Wang X, Guan H, Kong F, Xiao Z, Jing Y (2022). A vascularized conductive elastic patch for the repair of infarcted myocardium through functional vascular anastomoses and electrical integration. Adv Funct Mater.

[125] Kaufmann R, Theophile U (1967). Autonomously promoted extension effect in Purkinje fibers, papillary muscles and trabeculae carneae of rhesus monkeys. Pflugers Arch Gesamte Physiol Menschen Tiere.

[126] Fukada E, Yasuda I (1957). On the piezoelectric effect of bone. J Phys Soc Jpn.

[127] Kalinin SV, Rodriguez BJ, Shin J, Jesse S, Grichko V, Thundat T (2006). Bioelectromechanical imaging by scanning probe microscopy: Galvani's experiment at the nanoscale. Ultramicroscopy.

[128] Shamos MH, Lavine LS (1967). Piezoelectricity as a fundamental property of biological tissues. Nature.

[129] Anderson JC, Eriksson C (1970). Piezoelectric properties of dry and wet bone. Nature.

[130] Yucel T, Cebe P, Kaplan DL (2011). Structural origins of silk piezoelectricity. Adv Funct Mater.

[131] Denning D, Kilpatrick JI, Fukada E, Zhang N, Habelitz S, Fertala A (2017). Piezoelectric tensor of collagen fibrils determined at the nanoscale. ACS Biomater Sci Eng.

[132] Guerin S, Stapleton A, Chovan D, Mouras R, Gleeson M, Mckeown C (2018). Control of piezoelectricity in amino acids by supramolecular packing. Nat Mater.

[133] Lee BY, Zhang J, Zueger C, Chung W-J, Yoo SY, Wang E (2012). Virus-based piezoelectric energy generation. Nat Nanotechnol.

[134] Park IW, Kim KW, Hong Y, Yoon HJ, Lee Y, Gwak D (2020). Recent developments and prospects of M13-bacteriophage based piezoelectric energy harvesting devices. Nanomaterials.

[135] Yang F, Li J, Long Y, Zhang Z, Wang L, Sui J (2021). Wafer-scale heterostructured piezoelectric bio-organic thin films. Science.

[136] Shan G, Li X, Huang W (2020). AI-enabled wearable and flexible electronics for assessing full personal exposures. Innovation (N Y).

[137] Karan SK, Maiti S, Kwon O, Paria S, Maitra A, Si SK (2018). Nature driven spider silk as high energy conversion efficient bio-piezoelectric nanogenerator. Nano Energy.

[138] Karan SK, Maiti S, Paria S, Maitra A, Si SK, Kim JK (2018). A new insight towards eggshell membrane as high energy conversion efficient bio-piezoelectric energy harvester. Mater Today Energy.

[139] Wang X, Wang ZL, Yang Y (2016). Hybridized nanogenerator for simultaneously scavenging mechanical and thermal energies by electromagnetic-triboelectric-thermoelectric effects. Nano Energy.

[140] Sakaguchi M, Kashiwabara H (1992). A generation mechanism of triboelectricity due to the reaction of mechanoradicals with mechanoions which are produced by mechanical fracture of solid polymer. Colloid Polym Sci.

[141] Kim D, Jeon S-B, Kim JY, Seol M-L, Kim SO, Choi Y-K (2015). High-performance nanopattern triboelectric generator by block copolymer lithography. Nano Energy.

[142] Rajala S, Siponkoski T, Sarlin E, Mettänen M, Vuoriluoto M, Pammo A (2016). Cellulose nanofibril film as a piezoelectric sensor material. ACS Appl Mater Interfaces.

[143] Wang X, Zhang Y, Zhang X, Huo Z, Li X, Que M (2018). A highly stretchable transparent self-powered triboelectric tactile sensor with metallized nanofibers for wearable electronics. Adv Mater.

[144] Huang T, Zhang Y, He P, Wang G, Xia X, Ding G (2020). "self-matched" tribo/piezoelectric nanogenerators using vapor-induced phase-separated poly(vinylidene fluoride) and recombinant spider silk. Adv Mater.

[145] Chen FM, Liu X (2016). Advancing biomaterials of human origin for tissue engineering. Prog Polym Sci.

[146] Kim BS, Baez CE, Atala A (2000). Biomaterials for tissue engineering. World J Urol.

[147] Hubbell JA (1995). Biomaterials in tissue engineering. Biotechnology (N Y).

[148] Gong JP (2010). Why are double network hydrogels so tough?. Soft Matter.

[149] Lin F, Lu X, Wang Z, Lu Q, Lin G, Huang B (2019). In situ polymerization approach to cellulose–polyacrylamide interpenetrating network hydrogel with high strength and pH-responsive properties. Cellulose.

[150] Yang Y, Wang X, Yang F, Wang L, Wu D (2018). Highly elastic and ultratough hybrid ionic-covalent hydrogels with tunable structures and mechanics. Adv Mater.

[151] Tjong SC (2007). Novel nanoparticle-reinforced metal matrix composites with enhanced mechanical properties. Adv Eng Mater.

[152] Itoh H, Aso Y, Furuse M, Noishiki Y, Miyata T (2001). A honeycomb collagen carrier for cell culture as a tissue engineering scaffold. Artif Organs.

[153] Kakudo N, Shimotsuma A, Miyake S, Kushida S, Kusumoto K (2008). Bone tissue engineering using human adipose-derived stem cells and honeycomb collagen scaffold. J Biomed Mater Res A.

[154] Tang Z, Wang Y, Podsiadlo P, Kotov NA (2006). Biomedical applications of layer-by-layer assembly: from biomimetics to tissue engineering. Adv Mater.

[155] Shin K, Acri T, Geary S, Salem AK (2017). Biomimetic mineralization of biomaterials using simulated body fluids for bone tissue engineering and regenerative medicine. Tissue Eng Part A.

[156] Luo Y, Lode A, Wu C, Chang J, Gelinsky M (2015). Alginate/nanohydroxyapatite scaffolds with designed core/shell structures fabricated by 3D plotting and in situ mineralization for bone tissue engineering. ACS Appl Mater Interfaces.

[157] Chaudhuri O, Cooper-White J, Janmey PA, Mooney DJ, Shenoy VB (2020). Effects of extracellular matrix viscoelasticity on cellular behaviour. Nature.

[158] Mckinnon DD, Domaille DW, Cha JN, Anseth KS (2014). Biophysically defined and cytocompatible covalently adaptable networks as viscoelastic 3D cell culture systems. Adv Mater.

[159] Tang S, Ma H, Tu H-C, Wang H-R, Lin P-C, Anseth KS (2018). Adaptable fast relaxing boronate-based hydrogels for probing cell-matrix interactions. Adv Sci (Weinh).

[160] Brown TE, Carberry BJ, Worrell BT, Dudaryeva OY, Mcbride MK, Bowman CN (2018). Photopolymerized dynamic hydrogels with tunable viscoelastic properties through thioester exchange. Biomaterials.

[161] Marozas IA, Anseth KS, Cooper-White JJ (2019). Adaptable boronate ester hydrogels with tunable viscoelastic spectra to probe timescale dependent mechanotransduction. Biomaterials.

[162] Lou J, Stowers R, Nam S, Xia Y, Chaudhuri O (2018). Stress relaxing hyaluronic acid-collagen hydrogels promote cell spreading, fiber remodeling, and focal adhesion formation in 3D cell culture. Biomaterials.

[163] Loebel C, Mauck RL, Burdick JA (2019). Local nascent protein deposition and remodelling guide mesenchymal stromal cell mechanosensing and fate in three-dimensional hydrogels. Nat Mater.

[164] Chaudhuri O, Gu L, Klumpers D, Darnell M, Bencherif SA, Weaver JC (2016). Hydrogels with tunable stress relaxation regulate stem cell fate and activity. Nat Mater.

[165] Dooling LJ, Buck ME, Zhang W-B, Tirrell DA (2016). Programming molecular association and viscoelastic behavior in protein networks. Adv Mater.

[166] Kim J, Zhang G, Shi M, Suo Z (2021). Fracture, fatigue, and friction of polymers in which entanglements greatly outnumber cross-links. Science.

[167] Lin S, Liu X, Liu J, Yuk H, Loh H-C, Parada GA (2019). Anti-fatigue-fracture hydrogels. Sci Adv.

[168] Wang Z, Xiang C, Yao X, Le Floch P, Mendez J, Suo Z (2019). Stretchable materials of high toughness and low hysteresis. Proc Natl Acad Sci U S A.

[169] Kruzic JJ (2009). Materials science. Predicting fatigue failures. Science.

[170] Bi B, Liu H, Kang W, Zhuo R, Jiang X (2019). An injectable enzymatically crosslinked tyramine-modified carboxymethyl chitin hydrogel for biomedical applications. Colloids Surf B Biointerfaces.

[171] Ren K, Li B, Xu Q, Xiao C, He C, Li G (2017). Enzymatically crosslinked hydrogels based on linear poly(ethylene glycol) polymer: performance and mechanism. Polym Chem.

[172] Lee KY, Rowley JA, Eiselt P, Moy EM, Bouhadir KH, Mooney DJ (2000). Controlling mechanical and swelling properties of alginate hydrogels independently by cross-linker type and cross-linking density. Macromolecules.

[173] Glassman MJ, Chan J, Olsen BD (2013). Reinforcement of shear thinning protein hydrogels by responsive block copolymer self-assembly. Adv Funct Mater.

[174] Temenoff JS, Mikos AG (2000). Injectable biodegradable materials for orthopedic tissue engineering. Biomaterials.

[175] Deng Y, Hussain I, Kang M, Li K, Yao F, Liu S (2018). Self-recoverable and mechanical-reinforced hydrogel based on hydrophobic interaction with self-healable and conductive properties. Chem Eng J.

[176] Thornton PD, Mart RJ, Ulijn RV (2007). Enzyme-responsive polymer hydrogel particles for controlled release. Adv Mater.

[177] Fan H, Guo Z (2020). Bioinspired surfaces with wettability: biomolecule adhesion behaviors. Biomater Sci.

[178] Hancock MJ, Piraino F, Camci-Unal G, Rasponi M, Khademhosseini A (2011). Anisotropic material synthesis by capillary flow in a fluid stripe. Biomaterials.

[179] Hancock MJ, Yanagawa F, Jang Y-H, He J, Kachouie NN, Kaji H (2012). Designer hydrophilic regions regulate droplet shape for controlled surface patterning and 3D microgel synthesis. Small.

[180] Kryuchkov M, Bilousov O, Lehmann J, Fiebig M, Katanaev VL (2020). Reverse and forward engineering of Drosophila corneal nanocoatings. Nature.

[181] Wu J, Pan Z, Zhao ZY, Wang MH, Dong L, Gao HL (2022). Anti-swelling, robust, and adhesive extracellular matrix-mimicking hydrogel used as intraoral dressing. Adv Mater.

[182] Wang X, Yu Y, Yang C, Shao C, Shi K, Shang L (2021). Microfluidic 3D printing responsive scaffolds with biomimetic enrichment channels for bone regeneration. Adv Funct Mater.

[183] Revzin A, Russell RJ, Yadavalli VK, Koh WG, Deister C, Hile DD (2001). Fabrication of poly(ethylene glycol) hydrogel microstructures using photolithography. Langmuir.

[184] Liao W, Xiao Y, Gu Z, Li L, Yu X (2014). Preparation and properties of plasma sprayed strontium-doped calcium polyphosphate coating for bone tissue engineering. Ceram Int.

[185] Lu T, Qiao Y, Liu X (2012). Surface modification of biomaterials using plasma immersion ion implantation and deposition. Interface Focus.

[186] Castilho M, van Mil A, Maher M, Metz CHG, Hochleitner G, Groll J (2018). Melt electrowriting allows tailored microstructural and mechanical design of scaffolds to advance functional human myocardial tissue formation. Adv Funct Mater.

[187] Castilho M, Feyen D, Flandes-Iparraguirre M, Hochleitner G, Groll J, Doevendans PF (2017). Melt electrospinning writing of poly-hydroxymethylglycolide-co-ε-caprolactone-based scaffolds for cardiac tissue engineering. Adv Healthc Mater.

[188] Hou H, Hu K, Lin H, Forth J, Zhang W, Russell TP (2018). Reversible surface patterning by dynamic crosslink gradients: controlling buckling in 2D. Adv Mater.

[189] Hou H, Yin J, Jiang X (2019). Smart patterned surface with dynamic wrinkles. Acc Chem Res.

[190] Baudequin T, Tabrizian M (2018). Multilineage constructs for scaffold-based tissue engineering: a review of tissue-specific challenges. Adv Healthc Mater.

[191] Ma A, Chen H, Cui Y, Luo Z, Liang R, Wu Z (2019). Metalloporphyrin complex-based nanosonosensitizers for deep-tissue tumor theranostics by noninvasive sonodynamic therapy. Small.

[192] Rittikulsittichai S, Kolhatkar AG, Sarangi S, Vorontsova MA, Vekilov PG, Brazdeikis A (2016). Multi-responsive hybrid particles: thermo-, pH-, photo-, and magneto-responsive magnetic hydrogel cores with gold nanorod optical triggers. Nanoscale.

[193] Ke X, Coady DJ, Yang C, Engler AC, Hedrick JL, Yang YY (2014). pH-sensitive polycarbonate micelles for enhanced intracellular release of anticancer drugs: a strategy to circumvent multidrug resistance. Polym Chem.

[194] Hou H, Yin J, Jiang X (2016). Reversible Diels-alder reaction to control wrinkle patterns: from dynamic chemistry to dynamic patterns. Adv Mater.

[195] Li Z, Li Y, Chen C, Cheng Y (2021). Magnetic-responsive hydrogels: from strategic design to biomedical applications. J Control Release.

[196] Tang J, Qiao Y, Chu Y, Tong Z, Zhou Y, Zhang W (2019). Magnetic double-network hydrogels for tissue hyperthermia and drug release. J Mater Chem B Mater Biol Med.

[197] Gang F, Yan H, Ma C, Jiang L, Gu Y, Liu Z (2019). Robust magnetic double-network hydrogels with self-healing, MR imaging, cytocompatibility and 3D printability. Chem Commun (Camb).

[198] Zlotnick HM, Clark AT, Gullbrand SE, Carey JL, Cheng XM, Mauck RL (2020). Magneto-driven gradients of diamagnetic objects for engineering complex tissues. Adv Mater.

[199] Moncion A, Arlotta KJ, Kripfgans OD, Fowlkes JB, Carson PL, Putnam AJ (2016). Design and characterization of fibrin-based acoustically responsive scaffolds for tissue engineering applications. Ultrasound Med Biol.

[200] Gu Y, Zhong Y, Meng F, Cheng R, Deng C, Zhong Z (2013). Acetal-linked paclitaxel prodrug micellar nanoparticles as a versatile and potent platform for cancer therapy. Biomacromol.

[201] Zou J, Jafr G, Themistou E, Yap Y, Wintrob ZP, Alexandridis P (2011). pH-Sensitive brush polymer-drug conjugates by ring-opening metathesis copolymerization. Chem Commun (Camb).

[202] Zou J, Zhang F, Zhang S, Pollack SF, Elsabahy M, Fan J (2014). Poly(ethylene oxide)-block-polyphosphoester-graft-paclitaxel conjugates with acid-labile linkages as a pH-sensitive and functional nanoscopic platform for paclitaxel delivery. Adv Healthc Mater.

[203] Gan Q, Zhu J, Yuan Y, Liu H, Qian J, Li Y (2015). A dual-delivery system of pH-responsive chitosan-functionalized mesoporous silica nanoparticles bearing BMP-2 and dexamethasone for enhanced bone regeneration. J Mater Chem B Mater Biol Med.

[204] Abandansari HS, Ghanian MH, Varzideh F, Mahmoudi E, Rajabi S, Taheri P (2018). In situ formation of interpenetrating polymer network using sequential thermal and click crosslinking for enhanced retention of transplanted cells. Biomaterials.

[205] Rodell CB, Dusaj NN, Highley CB, Burdick JA (2016). Injectable and cytocompatible tough double-network hydrogels through tandem supramolecular and covalent crosslinking. Adv Mater.

[206] Mizrahi B, Shankarappa SA, Hickey JM, Dohlman JC, Timko BP, Whitehead KA (2013). A stiff injectable biodegradable elastomer. Adv Funct Mater.

[207] Rodell CB, Macarthur JW, Dorsey SM, Wade RJ, Wang LL, Woo YJ (2015). Shear-thinning supramolecular hydrogels with secondary autonomous covalent crosslinking to modulate viscoelastic properties in vivo. Adv Funct Mater.

[208] Desmet CM, Préat V, Gallez B (2018). Nanomedicines and gene therapy for the delivery of growth factors to improve perfusion and oxygenation in wound healing. Adv Drug Deliv Rev.

[209] Kim J, Nguyen TTH, Jin J, Septiana I, Son G-M, Lee G-H (2019). Anti-cariogenic characteristics of rubusoside. Biotechnol Bioprocess Eng.

[210] Kim S-E, Lee PW, Pokorski JK (2017). Biologically triggered delivery of EGF from polymer fiber patches. ACS Macro Lett.

[211] Khademhosseini A, Langer R, Borenstein J, Vacanti JP (2006). Microscale technologies for tissue engineering and biology. Proc Natl Acad Sci U S A.

[212] Hu C, Chu C, Liu L, Wang C, Jin S, Yang R (2021). Dissecting the microenvironment around biosynthetic scaffolds in murine skin wound healing. Sci Adv.

[213] Nikolova MP, Chavali MS (2019). Recent advances in biomaterials for 3D scaffolds: a review. Bioact Mater.

[214] Liu W, Sun Q, Zheng ZL, Gao YT, Zhu GY, Wei Q (2022). Topographic cues guiding cell polarization via distinct cellular mechanosensing pathways. Small.

[215] Chua JS, Chng CP, Moe AAK, Tann JY, Goh ELK, Chiam KH (2014). Extending neurites sense the depth of the underlying topography during neuronal differentiation and contact guidance. Biomaterials.

[216] Chen XQ, Chen XN, Zhu XD, Cai B, Fan HS, Zhang XD (2013). Effect of surface topography of hydroxyapatite on human osteosarcoma MG-63 cell: effect of surface topography of hydroxyapatite on human osteosarcoma MG-63 cell. Wuji Cailiao Xuebao (J Inorganic Mater).

[217] Zhao C, Xia L, Zhai D, Zhang N, Liu J, Fang B (2015). Designing ordered micropatterned hydroxyapatite bioceramics to promote the growth and osteogenic differentiation of bone marrow stromal cells. J Mater Chem B Mater Biol Med.

[218] Zhao Z, Li G, Ruan H, Chen K, Cai Z, Lu G (2021). Capturing magnesium ions via microfluidic hydrogel microspheres for promoting cancellous bone regeneration. ACS Nano.

[219] Yang J, Liang J, Zhu Y, Hu M, Deng L, Cui W (2021). Fullerol-hydrogel microfluidic spheres for in situ redox regulation of stem cell fate and refractory bone healing. Bioact Mater.

[220] Bian J, Cai F, Chen H, Tang Z, Xi K, Tang J (2021). Modulation of local overactive inflammation via injectable hydrogel microspheres. Nano Lett.

[221] Dai Y, Gao Z, Ma L, Wang D, Gao C (2016). Cell-free HA-MA/PLGA scaffolds with radially oriented pores for in situ inductive regeneration of full thickness cartilage defects. Macromol Biosci.

[222] Chen S, Wang H, Mainardi VL, Talò G, Mccarthy A, John JV (2021). Biomaterials with structural hierarchy and controlled 3D nanotopography guide endogenous bone regeneration. Sci Adv.

[223] Chen S, Mccarthy A, John JV, Su Y, Xie J (2020). Converting 2D nanofiber membranes to 3D hierarchical assemblies with structural and compositional gradients regulates cell behavior. Adv Mater.

[224] Yu Y, Shang L, Guo J, Wang J, Zhao Y (2018). Design of capillary microfluidics for spinning cell-laden microfibers. Nat Protoc.

[225] Rnjak-Kovacina J, Wise SG, Li Z, Maitz PKM, Young CJ, Wang Y (2012). Electrospun synthetic human elastin:collagen composite scaffolds for dermal tissue engineering. Acta Biomater.

[226] Zhang Y, Choi S-W, Xia Y (2012). Modifying the pores of an inverse opal scaffold with chitosan microstructures for truly three-dimensional cell culture. Macromol Rapid Commun.

[227] Shao C, Liu Y, Chi J, Wang J, Zhao Z, Zhao Y (2019). Responsive inverse opal scaffolds with biomimetic enrichment capability for cell culture. Research (Wash D C).

[228] Wang H, Zhao Z, Liu Y, Shao C, Bian F, Zhao Y (2018). Biomimetic enzyme cascade reaction system in microfluidic electrospray microcapsules. Sci Adv.

[229] Wang H, Xu Q, Shang L, Wang J, Rong F, Gu Z (2016). Boronate affinity molecularly imprinted inverse opal particles for multiple label-free bioassays. Chem Commun (Camb).

[230] Zhang YS, Zhu C, Xia Y (2017). Inverse opal scaffolds and their biomedical applications. Adv Mater.

[231] Osathanon T, Giachelli CM, Somerman MJ (2009). Immobilization of alkaline phosphatase on microporous nanofibrous fibrin scaffolds for bone tissue engineering. Biomaterials.

[232] Chen S, Carlson MA, Li X, Siddique A, Zhu W, Xie J (2021). Minimally invasive delivery of 3D shape recoverable constructs with ordered structures for tissue repair. ACS Biomater Sci Eng.

[233] Wan X, Liu S, Xin X, Li P, Dou J, Han X (2020). S-nitrosated keratin composite mats with NO release capacity for wound healing. Chem Eng J.

[234] Zhao X-H, Niu Y-N, Mi C-H, Gong H-L, Yang X-Y, Cheng J-S-Y (2021). Electrospinning nanofibers of microbial polyhydroxyalkanoates for applications in medical tissue engineering. J Polym Sci.

[235] Zhou Y, Yao H, Wang J, Wang D, Liu Q, Li Z (2015). Greener synthesis of electrospun collagen/hydroxyapatite composite fibers with an excellent microstructure for bone tissue engineering. Int J Nanomed.

[236] Zhu S, Zeng W, Meng Z, Luo W, Ma L, Li Y (2019). Using wool keratin as a basic resist material to fabricate precise protein patterns. Adv Mater.

[237] Zhu S, Tang Y, Lin C, Liu XY, Lin Y (2021). Recent advances in patterning natural polymers: from nanofabrication techniques to applications. Small Methods.

[238] Hou H, Li F, Su Z, Yin J, Jiang X (2017). Light-reversible hierarchical patterns by dynamic photo-dimerization induced wrinkles. J Mater Chem C Mater Opt Electron Devices.

[239] Zorlutuna P, Annabi N, Camci-Unal G, Nikkhah M, Cha JM, Nichol JW (2012). Microfabricated biomaterials for engineering 3D tissues. Adv Mater.

[240] Moffa M, Sciancalepore AG, Passione LG, Pisignano D (2014). Combined nano- and micro-scale topographic cues for engineered vascular constructs by electrospinning and imprinted micro-patterns. Small.

[241] Wang H, Cai L, Zhang D, Shang L, Zhao Y (2021). Responsive Janus structural color hydrogel micromotors for label-free multiplex assays. Research (Wash D C).

[242] Wang H, Zhang H, Zhang D, Wang J, Tan H, Kong T (2021). Enzyme-functionalized structural color hydrogel particles for urea detection and elimination. J Clean Prod.

[243] Luo Z, Che J, Sun L, Yang L, Zu Y, Wang H (2021). Microfluidic electrospray photo-crosslinkable κ-Carrageenan microparticles for wound healing. Engin Regen.

[244] Wei X, Bian F, Zhang H, Wang H, Zhu Y (2021). Multiplex assays of bladder cancer protein markers with magnetic structural color hydrogel microcarriers based on microfluidics. Sens Actuators B Chem.

[245] Shang L, Cheng Y, Zhao Y (2017). Emerging droplet microfluidics. Chem Rev.

[246] Yu Y, Chen G, Guo J, Liu Y, Ren J, Kong T (2018). Vitamin metal–organic framework-laden microfibers from microfluidics for wound healing. Mater Horiz.

[247] Yang L, Liu Y, Sun L, Zhao C, Chen G, Zhao Y (2021). Biomass microcapsules with stem cell encapsulation for bone repair. Nanomicro Lett.

[248] Pedde RD, Mirani B, Navaei A, Styan T, Wong S, Mehrali M (2017). Emerging biofabrication strategies for engineering complex tissue constructs. Adv Mater.

[249] Wang X, Yang C, Yu Y, Zhao Y (2022). In situ 3D bioprinting living photosynthetic scaffolds for autotrophic wound healing. Research (Wash D C).

[250] Boland T, Tao X, Damon BJ, Manley B, Kesari P, Jalota S (2007). Drop-on-demand printing of cells and materials for designer tissue constructs. Mater Sci Eng C Mater Biol Appl.

[251] Wang H, Liu Y, Chen Z, Sun L, Zhao Y (2020). Anisotropic structural color particles from colloidal phase separation. Sci Adv.

[252] Hu Y, Zhang H, Wei H, Cheng H, Cai J, Chen X (2022). Scaffolds with anisotropic structure for neural tissue engineering. Eng Regener.

[253] Zhang H, Zhang H, Wang H, Zhao Y, Chai R (2022). Natural proteins-derived asymmetric porous conduit for peripheral nerve regeneration. Appl Mater Today.

[254] Kong B, Sun L, Liu R, Chen Y, Shang Y, Tan H (2022). Recombinant human collagen hydrogels with hierarchically ordered microstructures for corneal stroma regeneration. Chem Eng J.

[255] Hou H, Gan Y, Jiang X, Yin J (2017). Facile and robust strategy to antireflective photo-curing coating through self-wrinkling. Chin Chem Lett.

[256] Hou H, Gan Y, Yin J, Jiang X (2014). Multifunctional POSS-based nano-photo-initiator for overcoming the oxygen inhibition of photo-polymerization and for creating self-wrinkled patterns. Adv Mater Interfaces.

[257] Hou H, Gan Y, Yin J, Jiang X (2017). Polymerization-induced growth of microprotuberance on the photocuring coating. Langmuir.

[258] Makvandi P, Maleki A, Shabani M, Hutton ARJ, Kirkby M, Jamaledin R (2022). Bioinspired microneedle patches: biomimetic designs, fabrication, and biomedical applications. Matter.

[259] Makvandi P, Jamaledin R, Chen G, Baghbantaraghdari Z, Zare EN, Di Natale C (2021). Stimuli-responsive transdermal microneedle patches. Mater Today (Kidlington).

[260] Schneider JP, Pochan DJ, Ozbas B, Rajagopal K, Pakstis L, Kretsinger J (2002). Responsive hydrogels from the intramolecular folding and self-assembly of a designed peptide. J Am Chem Soc.

[261] Moore AN, Hartgerink JD (2017). Self-assembling multidomain peptide nanofibers for delivery of bioactive molecules and tissue regeneration. Acc Chem Res.

[262] Kumar VA, Taylor NL, Shi S, Wang BK, Jalan AA, Kang MK (2015). Highly angiogenic peptide nanofibers. ACS Nano.

[263] L'Heureux N, Pâquet S, Labbé R, Germain L, Auger FA (1998). A completely biological tissue-engineered human blood vessel. FASEB J.

[264] Capuana E, Lopresti F, Carfì Pavia F, Brucato V, La Carrubba V (2021). Solution-based processing for scaffold fabrication in tissue engineering applications: a brief review. Polymers (Basel).

[265] Grenier J, Duval H, Barou F, Lv P, David B, Letourneur D (2019). Mechanisms of pore formation in hydrogel scaffolds textured by freeze-drying. Acta Biomater.

[266] Wang L, Jiang J, Hua W, Darabi A, Song X, Song C (2016). Mussel-inspired conductive cryogel as cardiac tissue patch to repair myocardial infarction by migration of conductive nanoparticles. Adv Funct Mater.

[267] Stokols S, Tuszynski MH (2004). The fabrication and characterization of linearly oriented nerve guidance scaffolds for spinal cord injury. Biomaterials.

[268] Wang RM, Christman KL (2016). Decellularized myocardial matrix hydrogels: in basic research and preclinical studies. Adv Drug Deliv Rev.

[269] Ott HC, Matthiesen TS, Goh S-K, Black LD, Kren SM, Netoff TI (2008). Perfusion-decellularized matrix: using nature's platform to engineer a bioartificial heart. Nat Med.

[270] Uygun BE, Soto-Gutierrez A, Yagi H, Izamis M-L, Guzzardi MA, Shulman C (2010). Organ reengineering through development of a transplantable recellularized liver graft using decellularized liver matrix. Nat Med.

[271] Xu F, Kang T, Deng J, Liu J, Chen X, Wang Y (2020). Functional nanoparticles activate a decellularized liver scaffold for blood detoxification. Small.

[272] Bousalis D, McCrary MW, Vaughn N, Hlavac N, Evering A, Kolli S (2022). Decellularized peripheral nerve as an injectable delivery vehicle for neural applications. J Biomed Mater Res A.

[273] Saldin LT, Cramer MC, Velankar SS, White LJ, Badylak SF (2017). Extracellular matrix hydrogels from decellularized tissues: structure and function. Acta Biomater.

[274] Singelyn JM, DeQuach JA, Seif-Naraghi SB, Littlefield RB, Schup-Magoffin PJ, Christman KL (2009). Naturally derived myocardial matrix as an injectable scaffold for cardiac tissue engineering. Biomaterials.

[275] Hong JY, Seo Y, Davaa G, Kim H-W, Kim SH, Hyun JK (2020). Decellularized brain matrix enhances macrophage polarization and functional improvements in rat spinal cord injury. Acta Biomater.

[276] Wolf MT, Daly KA, Brennan-Pierce EP, Johnson SA, Carruthers CA, D'amore A (2012). A hydrogel derived from decellularized dermal extracellular matrix. Biomaterials.

[277] Collins MN, Ren G, Young K, Pina S, Reis RL, Oliveira JM (2021). Scaffold fabrication technologies and structure/function properties in bone tissue engineering. Adv Funct Mater.

[278] Lepedda AJ, Nieddu G, Formato M, Baker MB, Fernández-Pérez J, Moroni L (2021). Glycosaminoglycans: from vascular physiology to tissue engineering applications. Front Chem.

[279] Rho KS, Jeong L, Lee G, Seo B-M, Park YJ, Hong S-D (2006). Electrospinning of collagen nanofibers: effects on the behavior of normal human keratinocytes and early-stage wound healing. Biomaterials.

[280] Ahn S, Yoon H, Kim G, Kim Y, Lee S, Chun W (2010). Designed three-dimensional collagen scaffolds for skin tissue regeneration. Tissue Eng Part C Methods.

[281] Ng WL, Goh MH, Yeong WY, Naing MW (2018). Applying macromolecular crowding to 3D bioprinting: fabrication of 3D hierarchical porous collagen-based hydrogel constructs. Biomater Sci.

[282] Maghdouri-White Y, Sori N, Petrova S, Wriggers H, Kemper N, Dasgupta A (2021). Biomanufacturing organized collagen-based microfibers as a Tissue engineered device (TEND) for tendon regeneration. Biomed Mater.

[283] Inzana JA, Olvera D, Fuller SM, Kelly JP, Graeve OA, Schwarz EM (2014). 3D printing of composite calcium phosphate and collagen scaffolds for bone regeneration. Biomaterials.

[284] Yu L, Rowe DW, Perera IP, Zhang J, Suib SL, Xin X (2020). Intrafibrillar mineralized collagen-hydroxyapatite-based scaffolds for bone regeneration. ACS Appl Mater Interfaces.

[285] Mirkhalaf M, Goldsmith J, Ren J, Dao A, Newman P, Schindeler A (2021). Highly substituted calcium silicates 3D printed with complex architectures to produce stiff, strong and bioactive scaffolds for bone regeneration. Appl Mater Today.

[286] Yang C, Wang X, Ma B, Zhu H, Huan Z, Ma N (2017). 3D-printed bioactive Ca3SiO5 bone cement scaffolds with nano surface structure for bone regeneration. ACS Appl Mater Interfaces.

[287] Bao W, Li M, Yang Y, Wan Y, Wang X, Bi N (2020). Advancements and frontiers in the high performance of natural hydrogels for cartilage tissue engineering. Front Chem.

[288] Little CJ, Kulyk WM, Chen X (2014). The effect of chondroitin sulphate and hyaluronic acid on chondrocytes cultured within a fibrin-alginate hydrogel. J Funct Biomater.

[289] Ko C-S, Huang J-P, Huang C-W, Chu IM (2009). Type II collagen-chondroitin sulfate-hyaluronan scaffold cross-linked by genipin for cartilage tissue engineering. J Biosci Bioeng.

[290] Kang E, Jeong GS, Choi YY, Lee KH, Khademhosseini A, Lee S-H (2011). Digitally tunable physicochemical coding of material composition and topography in continuous microfibres. Nat Mater.

[291] Reversat A, Gaertner F, Merrin J, Stopp J, Tasciyan S, Aguilera J (2020). Cellular locomotion using environmental topography. Nature.

[292] Dalby MJ, Gadegaard N, Oreffo ROC (2014). Harnessing nanotopography and integrin-matrix interactions to influence stem cell fate. Nat Mater.

[293] Sun L, Gao W, Fu X, Shi M, Xie W, Zhang W (2018). Enhanced wound healing in diabetic rats by nanofibrous scaffolds mimicking the basketweave pattern of collagen fibrils in native skin. Biomater Sci.

[294] Chen W, Ma J, Zhu L, Morsi Y, Ei-Hamshary H, Al-Deyab SS (2016). Superelastic, superabsorbent and 3D nanofiber-assembled scaffold for tissue engineering. Colloids Surf B Biointerfaces.

[295] Tognetti L, Pianigiani E, Ierardi F, Lorenzini G, Casella D, Liso FG (2021). The use of human acellular dermal matrices in advanced wound healing and surgical procedures: state of the art. Dermatol Ther.

[296] Choi JS, Lee SJ, Christ GJ, Atala A, Yoo JJ (2008). The influence of electrospun aligned poly(ɛ-caprolactone)/collagen nanofiber meshes on the formation of self-aligned skeletal muscle myotubes. Biomaterials.

[297] Kurpinski KT, Stephenson JT, Janairo RRR, Lee H, Li S (2010). The effect of fiber alignment and heparin coating on cell infiltration into nanofibrous PLLA scaffolds. Biomaterials.

[298] Xie J, Macewan MR, Ray WZ, Liu W, Siewe DY, Xia Y (2010). Radially aligned, electrospun nanofibers as dural substitutes for wound closure and tissue regeneration applications. ACS Nano.

[299] Chen S, Wang H, Mccarthy A, Yan Z, Kim HJ, Carlson MA (2019). Three-dimensional objects consisting of hierarchically assembled nanofibers with controlled alignments for regenerative medicine. Nano Lett.

[300] Xie J, Li X, Lipner J, Manning CN, Schwartz AG, Thomopoulos S (2010). "Aligned-to-random" nanofiber scaffolds for mimicking the structure of the tendon-to-bone insertion site. Nanoscale.

[301] Dapporto M, Sprio S, Fabbi C, Figallo E, Tampieri A (2016). A novel route for the synthesis of macroporous bioceramics for bone regeneration. J Eur Ceram Soc.

[302] John JV, McCarthy A, Wang H, Luo Z, Li H, Wang Z (2021). Freeze-casting with 3D-printed templates creates anisotropic microchannels and patterned macrochannels within biomimetic nanofiber aerogels for rapid cellular infiltration. Adv Healthc Mater.

[303] Oh SH, Kim TH, Im GI, Lee JH (2010). Investigation of pore size effect on chondrogenic differentiation of adipose stem cells using a pore size gradient scaffold. Biomacromol.

[304] Zhang Q, Lu H, Kawazoe N, Chen G (2013). Preparation of collagen porous scaffolds with a gradient pore size structure using ice particulates. Mater Lett.

[305] Xu H, Holzwarth JM, Yan Y, Xu P, Zheng H, Yin Y (2014). Conductive PPY/PDLLA conduit for peripheral nerve regeneration. Biomaterials.

[306] Wu DT, Jeffreys N, Diba M, Mooney DJ (2022). Viscoelastic biomaterials for tissue regeneration. Tissue Eng Part C Methods.

[307] Costa JB, Park J, Jorgensen AM, Silva-Correia J, Reis RL, Oliveira JM (2020). 3D bioprinted highly elastic hybrid constructs for advanced fibrocartilaginous tissue regeneration. Chem Mater.

[308] Davenport Huyer L, Zhang B, Korolj A, Montgomery M, Drecun S, Conant G (2016). Highly elastic and moldable polyester biomaterial for cardiac tissue engineering applications. ACS Biomater Sci Eng.

[309] Bajpai AK, Shukla SK, Bhanu S, Kankane S (2008). Responsive polymers in controlled drug delivery. Prog Polym Sci.

[310] Chung HJ, Bae JW, Park HD, Lee JW, Park KD (2005). Thermosensitive chitosans as novel injectable biomaterials. Macromol Symp.

[311] Shi J, Yu L, Ding J (2021). PEG-based thermosensitive and biodegradable hydrogels. Acta Biomater.

[312] He J, Zhang Z, Yang Y, Ren F, Li J, Zhu S (2021). Injectable self-healing adhesive pH-responsive hydrogels accelerate gastric hemostasis and wound healing. Nanomicro Lett.

